# Differential Complex Formation via Paralogs in the Human Sin3 Protein Interaction Network

**DOI:** 10.1074/mcp.RA120.002078

**Published:** 2020-11-25

**Authors:** Mark K. Adams, Charles A.S. Banks, Janet L. Thornton, Cassandra G. Kempf, Ying Zhang, Sayem Miah, Yan Hao, Mihaela E. Sardiu, Maxime Killer, Gaye L. Hattem, Alexis Murray, Maria L. Katt, Laurence Florens, Michael P. Washburn

**Affiliations:** 1 Stowers Institute for Medical Research, Kansas City, Missouri, USA; 2 Department of Pathology & Laboratory Medicine, University of Kansas Medical Center, Kansas City, Kansas, USA

**Keywords:** Chromatin function or biology, epigenetics, protein complex analysis, pathway analysis, protein-protein interactions*, systems biology*, subcellular analysis, nuclear translocation, cross linking, DSSO, histone deacetylase, SIN3

## Abstract

Despite the continued analysis of HDAC inhibitors in clinical trials, the heterogeneous nature of the protein complexes they target limits our understanding of the beneficial and off-target effects associated with their application. Among the many HDAC protein complexes found within the cell, Sin3 complexes are conserved from yeast to humans and likely play important roles as regulators of transcriptional activity. The presence of two Sin3 paralogs in humans, SIN3A and SIN3B, may result in a heterogeneous population of Sin3 complexes and contributes to our poor understanding of the functional attributes of these complexes. Here, we profile the interaction networks of SIN3A and SIN3B to gain insight into complex composition and organization. In accordance with existing data, we show that Sin3 paralog identity influences complex composition. Additionally, chemical cross-linking MS identifies domains that mediate interactions between Sin3 proteins and binding partners. The characterization of rare SIN3B proteoforms provides additional evidence for the existence of conserved and divergent elements within human Sin3 proteins. Together, these findings shed light on both the shared and divergent properties of human Sin3 proteins and highlight the heterogeneous nature of the complexes they organize.

## Teaser

Humans possess two Sin3 paralogs, SIN3A and SIN3B, that serve as scaffolds within the Sin3 complexes. There is existing evidence that paralog identity influences Sin3 complex composition. Using proteomic profiling, we characterize this influence and highlight heterogeneity within the population of Sin3 complexes. We incorporate chemical cross-linking mass spectrometry into our assays to define domains within SIN3A and SIN3B that mediate protein interactions. These data highlight the modular nature of Sin3 complexes.

## Graphical Abstract

**Figure:**
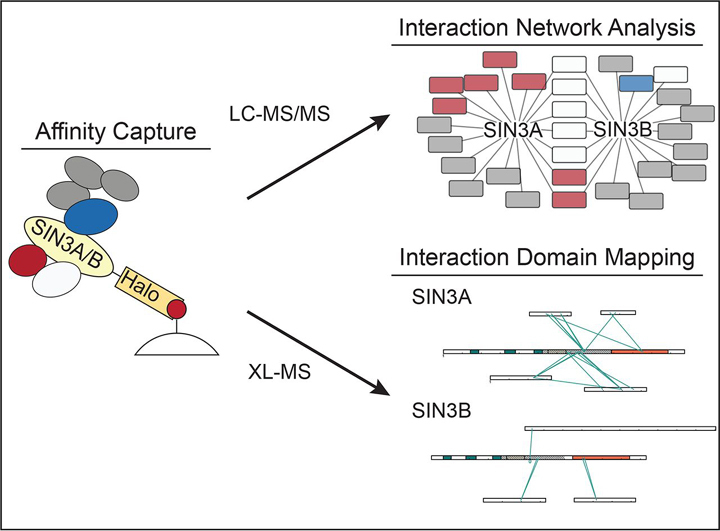

• Sin3 paralog identity influences Sin3 complex composition.
• Chemical cross-linking mass spectrometry identifies domains in SIN3A and SIN3B that mediate complex formation.
• Complex subunit homology to yeast Sin3 complex components may assist in defining distinct forms of the Sin3 complex in humans.
• A nuclear import signal within SIN3B is identified via chemical cross-linking mass spectrometry.

Over 13,000 or 70% of protein coding genes within the human genome have at least one paralog (1). The acquisition of additional copies of a gene through duplication events provides opportunities for the development of unique gene products with distinct regulatory mechanisms (2). Functional divergence can result from gene duplication and protein paralog identity can influence the composition of large protein complexes (3). However, the consequences of paralog switching are largely overlooked during the characterization of proteins, protein complexes, and protein interaction networks.

Classically associated with transcriptional repression, the removal of histone lysine acetyl groups by the Sin3 histone deacetylase (HDAC) complexes represents a central mechanism whereby transcriptional status is regulated (4). Named for the scaffolding protein of the complexes, Sin3 complexes are well studied in *Saccharomyces cerevisiae* (5, 6). However, in higher eukaryotes, the presence of additional components not found in lower eukaryotic forms of the Sin3 complexes likely increases the diversity of complex functions. Contributing to this expansion of components is the acquisition of paralogous genes encoding Sin3 proteins. The two Sin3 paralogs present within mammals, SIN3A and SIN3B, have undergone substantial divergence and maintain only 63% sequence similarity at the protein level in humans (supplemental Fig. S1).

There is accumulating evidence that SIN3A and SIN3B are not functionally redundant within mammals. It has been shown that SIN3A can act as a suppressor of metastasis, whereas SIN3B can act as a pro-metastatic factor (7). Additionally, genetic deletion of murine *Sin3a* results in early embryonic lethality whereas deletion of *Sin3b* induces late gestational lethality (8, 9). That SIN3A and SIN3B cannot compensate for the loss of one another provides evidence for paralog-specific functions within mammals and suggests that variations within the Sin3 complexes have functional consequences.

Although the mechanisms responsible for divergent influences on development as well as cancer cell metastatic potential remain poorly understood, there is growing evidence that Sin3 paralog identity influences Sin3 complex composition (10). Heterogeneity within a population of Sin3 complexes is not unprecedented as two distinct forms of the complex, known as Rpd3L (Sin3 large) and Rpd3S (Sin3 small) are found in *S. cerevisiae*. Whereas the ∼1.2 MDa Rpd3L complex localizes to gene promoter regions and influences transcription initiation, the ∼0.6 MDa Rpd3S complex is mostly found within actively transcribed genes and inhibits intragenic transcription (5, 6). These two protein complexes share a common core of proteins, consisting of Rpd3, Sin3, and Ume1 (11), but are differentiated by their unique sets of subunits.

Higher eukaryotes have genes encoding proteins that have homology with *S. cerevisiae* Sin3 complex components. Among proteins found in humans, HDAC1/HDAC2, SIN3A/SIN3B, and RBBP4/RBBP7 have homology to the *S. cerevisiae* core Sin3 complex components Rpd3, Sin3, and Ume1, respectively. In addition to possessing proteins that share homology with *S. cerevisiae* Sin3 core complex components, humans also have proteins that have homology to Rpd3L- and Rpd3S-specific components. SUDS3/BRMS1/BRMS1L, SAP30/SAP30L, and ING1/ING2 have homology to Rpd3L-specific components Sds3, Sap30, and Pho23, respectively (12, 13, 14). Components specifically found within Rpd3S, Rco1, and Eaf3, share homology with human PHF12 and MORF4L1, respectively (15, 16). Although SIN3A can clearly interact with Rpd3L component homologs, data supports the existence of SIN3B complexes that contain Rpd3S component homologs (17, 18). However, a combined analysis of Sin3 interaction partners to define modularity and identify mutual exclusivity within the network has not been performed.

Using a combination of shotgun proteomics and chemical cross-linking MS (XL-MS), we profile the Sin3 interaction network. Our results outline the influence of paralog switching on complex construction. These findings define direct interactions within the Sin3 interaction network and identify divergent properties of the Sin3 paralogs.

## EXPERIMENTAL PROCEDURES

##### Preparation of Expression Vectors and Expression in Flp-In™-293 Cell Lines

Expression vectors were prepared as described in Supplemental Methods. Stable cell lines were produced using Flp-In™-293 cells (Thermo Fisher Scientific, Waltham, MA), authenticated by STR profiling (FTA barcode: STR14169), and tested for mycoplasma using mycoplasma detection kits (American Type Culture Collection, Manassas, VA). The day before transfection, cells were plated at 50% confluency onto a 100 mm tissue culture plate containing DMEM and 10% FBS, then incubated at 37 °C in 5% CO_2_ overnight. The following day, cells were washed two times with Opti-MEM, then incubated with 8 ml Opti-MEM containing GlutaMAX supplement (Thermo Fisher Scientific). Plasmid DNA (4 µg total; 3.6 µg pOG44 + 0.4 µg DNA of interest) was added to 800 µL of Opti-MEM with GlutaMAX supplement along with 16 µL FuGENE® HD Transfection Reagent (Promega Corporation, Madison, WI), incubated for 15-30 min, then added dropwise to the prepared plate. One ml of FBS (Peak Serum, Inc, Wellington CO) was added the next morning. On day three of incubation, cells were split 1:10 and placed into selection media (DMEM/10% FBS/100 µg/ml Hygromycin B). Media was changed every 3 days for a total of three media changes. After 2 weeks, colonies were visible and picked for screening. Flp-In™-293 cell lines stably expressing HaloTag-SAP30, HaloTag-SAP30L, and HaloTag-SUDS3 were previously described (19, 20).

##### Fluorescence Microscopy

Flp-In™-293 cell lines stably expressing HaloTag® fusion proteins were seeded at 40% confluency in 35 mm MatTek glass bottom dishes (MatTek Corporation, Ashland, MA) containing DMEM supplemented with penicillin-streptomycin solution, GlutaMAX supplement, and FBS to a final concentration of 10%. Cell media was supplemented with HaloTag® TMRDirect™ Ligand (Promega Corporation) to a final concentration of 20 nm 16–24 h after seeding. Cells were then cultured for an additional 16–24 h. Hoechst 33258 solution (Sigma Aldrich Corporation, St. Louis, MO) was added to culture dishes 80 min before imaging.

Media conditions for transient transfection of 293T cells (American Type Culture Collection) with plasmid DNA were as stated for the imaging of the stable expression cell lines. Cells continued to grow 16–24 h after seeding at 40% confluency in 35 mm MatTek glass bottom dishes before transfection. Cells were transfected with Opti-MEM media containing 2.5 µg of plasmid, 5 µL LipofectAMINE™ LTX Reagent (Thermo Fisher Scientific), and 2.5 µL PLUS™ Reagent (Thermo Fisher Scientific). Cell media was supplemented with HaloTag® TMRDirect™ Ligand to a final concentration of 20 nm 16-24 h after transfection. After an additional 16–24 h of culture at 37 °C and 5% CO_2_, Hoechst 33258 solution was added to the culture dishes and incubation was continued for 1 h.

Cells were washed and imaged in Opti-MEM media. Images were captured on a PerkinElmer Life Sciences UltraVIEW VoX spinning disk microscope (PerkinElmer, Inc., Waltham, MA), Axiovert 200M base (Carl Zeiss AG, Oberkochen, Germany), or an inverted LSM-700 point scanning confocal microscope controlled by Zeiss Zen software (Carl Zeiss AG). A 40× plan-apochromat (NA 1.4) oil objective was used to acquire images when operating the LSM-700 microscope. Detection wavelength ranges were 300–483 nm for Hoechst and 570–800 nm for HaloTag® TMRDirect™ Ligand. SP 490 and LP 490 filter sets were employed when imaging Hoechst and HaloTag® TMRDirect™ Ligand, respectively, on the LSM-700 microscope.

##### Affinity Purification of Recombinant Proteins from Flp-In™-293 Cells for Multidimensional Protein Identification Technology (MudPIT) Analysis

Cells were lysed and recombinant proteins were isolated using Magne® HaloTag® Beads (Promega Corporation) as previously described (19). Briefly, 2 confluent 850 cm^2^ culture vessels of Flp-In™-293 cells stably expressing a transgene were lysed and incubated with HL-SAN nuclease (ArcticZymes, Tromsø, Norway) at a final concentration of 2 U/ml for 2 h at 4 °C before protein enrichment. Recombinant protein was isolated via incubation with Magne® HaloTag® Beads and eluted with AcTEV™ Protease (Thermo Fisher Scientific). Affinity purified (AP) proteins were TCA precipitated, digested with Endoproteinase Lys-C or Recombinant Endoproteinase LysC (Promega Corporation), then digested further with Sequencing Grade Trypsin (Promega Corporation). Peptides were loaded onto triphasic MudPIT microcapillary columns as previously described (21). Columns were placed in-line with an 1100 Series HPLC system (Agilent Technologies, Inc., Santa Clara, CA) coupled to a linear ion trap mass spectrometer (Thermo Fisher Scientific) and peptides were resolved using 10-step MudPIT chromatography as previously described (22).

##### Preparation of Samples for Chemical Cross-linking Mass Spectrometry

For each replicate, 3 confluent 850 cm^2^ culture vessels of Flp-In™-293 cells stably expressing SIN3A-HaloTag or SIN3B_2-HaloTag were harvested. Protein was enriched using Magne® HaloTag® Beads and cross-linked with disuccinimidyl sulfoxide (DSSO) as previously described (20). Briefly, DSSO (Cayman Chemical Company, Ann Arbor, MI) was added to samples to a final concentration of 5 mm while protein was immobilized on beads. Samples were incubated at room temperature for 40 min. Reactions were quenched with the addition of NH_4_HCO_3_ to a final concentration of 50 mm and samples were incubated an additional 15 min at room temperature. Recombinant proteins were eluted with AcTEV™ Protease at room temperature overnight. Proteins were TCA precipitated and digested as previously described (19). Peptides were resolved on a 50 μm inner diameter microcapillary column containing 15 cm of 1.9 μm C18 resin (ESI Source Solutions, Woburn, MA). Peptides were identified with an Orbitrap Fusion™ Lumos™ mass spectrometer (Thermo Fisher Scientific) and data were acquired as previously described (20).

##### Experimental Design and Statistical Rationale

To characterize protein interaction networks, a minimum of three biological replicates were acquired for each affinity purification MS (APMS) analysis. As a control, Flp-In™-293 cells expressing no transgenes were also analyzed. Acquired .RAW files were converted to .ms2 files using RAWDistiller (23). ProLuCID v1.3.5 (24) was used to match spectra against a database (Genome Reference Consortium Human Build 38 patch release 13) containing 44,519 unique proteins, 426 of which were contaminant proteins. The database was shuffled for false discovery rate (FDR) estimation, producing a final database that contained 89,038 total sequences. The database was searched for fully tryptic peptides, allowing for a maximum of 3 internal cleavage sites and a minimum peptide length of 7 amino acids. Database searches were performed with a static modification of +57 Da for cysteine, a dynamic modification of +16 Da for methionine, and a mass tolerance of 800 ppm for precursor and fragment ions. DTASelect and Contrast (25) were used to filter results and NSAF v7 (26) was used to calculate label-free quantitative dNSAF values and generate final reports (supplemental Tables S2A–2B and S4A–4B). The spectral FDR mean ± S.D. for the 70 MudPIT runs was 0.337% ± 0.138%, the mean ± S.D. peptide FDR was 0.254% ± 0.122%, and the mean ± S.D. protein FDR was 0.917% ± 0.405%. For the analysis of SIN3A and SIN3B isoforms, the spectral FDR mean ± S.D. for the 20 MudPIT runs was 0.282% ± 0.133%, the mean ± S.D. peptide FDR was 0.272% ± 0.097%, and the mean ± S.D. protein FDR was 0.874% ± 0.329%. A DTASelect filter also established a minimum peptide length of 7 amino acids, and proteins that were subsets of others were removed using the parsimony option in Contrast.

To identify cross-linked peptides, 5 technical replicates of SIN3B_2-HaloTag and 3 technical replicates for SIN3A-HaloTag were analyzed. Peptides were analyzed with an Orbitrap Fusion™ Lumos™ and data acquisition was performed as previously described (20). Briefly, cross-linked peptides were identified using Proteome Discoverer v2.4 and the XlinkX module (27). Acquired .RAW files were searched against a human proteome database (Genome Reference Consortium Human Build 38 patch release 13) containing 44,519 unique protein sequences, 426 of which were contaminant proteins. For XlinkX searches, the database was searched for fully tryptic peptides, allowing for a maximum of 2 missed cleavages and a minimum peptide length of 5 amino acids. Searches were performed with a static modification of +57.021 Da for cysteine and a dynamic modification of +15.995 Da for methionine. Precursor mass tolerance, FTMS fragment mass tolerance, and ITMS Fragment tolerance, were set to 10 ppm, 20 ppm, and 0.5 Da, respectively. Xlink Validator FDR threshold was set to 0.01. For Sequest HT searches, the database was searched for fully tryptic peptides, allowing for a maximum of 2 missed cleavages and a minimum peptide length of 6 amino acids. Searches were performed with a static modification of +57.021 Da for cysteine, a dynamic modification of +15.995 Da for methionine, a dynamic modification of +176.014 Da for lysine (water-quenched DSSO monoadduct), and a dynamic modification of + 279.08 Da for lysine (Tris-quenched DSSO monoadduct). Precursor mass tolerance and fragment mass tolerance were set to 10 ppm and 0.6 Da, respectively. Percolator target FDR (Strict) was set to 0.01. Identified cross-link spectrum matches are reported in supplemental Table S3.

Data that has been previously described was included in our analyses and is summarized in supplemental Table S1. All MS data has been deposited into the MassIVE repository (http://massive.ucsd.edu). Data set identifiers are supplied in supplemental Table S1.

##### Analysis of Proteomics Data Sets

To identify high-confidence interaction partners, QSPEC v1.3.5 (28) was used to calculate Z-statistic and log2 fold change values. Prey proteins that were not present in at least half of at least one bait protein purification (supplemental Table S2C, supplemental Table S4C) were excluded before QSPEC scoring (supplemental Tables S2D, S4D). QSPEC analysis was performed with a burn in value of 2000 and 10,000 iterations. To identify enriched proteins over negative AP controls, Z-statistic values of ≥ 3 and log2 fold change values ≥ 2 were selected as filter values.

##### Enzyme Activity Assays

HDAC activity assays of transiently produced proteins were performed as described (29). Briefly, ∼1 × 10^7^ 293T cells were plated in 150 mm dishes and cultured in 25 ml DMEM + 10% fetal bovine serum + 1 × GlutaMAX Supplement. 24 h after seeding, cells were transfected with 7.5 µg plasmid DNA, 7.5 µL Plus Reagent, and 50 µL LipofectAMINE LTX diluted in 6.6 ml OptiMEM. Cells were harvested after an additional 48 h of culture. Two mg of whole cell extract were added to 100 µL of washed Magne® HaloTag® Beads slurry and incubated at 4 °C for 2 h. Beads were washed 4 times with 1 ml cold TBS pH 7.4 + 0.05% Igepal CA-630 (Sigma Aldrich Corporation). Protein was eluted with 5 units AcTEV™ Protease (Thermo Fisher Scientific) in 100 µL of 50 mm Tris-HCl pH 8.0, 0.5 mm EDTA, 1 mm DTT for 16 h at 4 °C. Ten µL of the 100 µL purified protein was diluted with 32.5 µL TBS (25 mm Tris, 150 mm NaCl, 2 mm KCl, pH 7.4). Samples were supplemented with 2.5 µL of DMSO or 200 μm SAHA (Cayman Chemical Company) resuspended in DMSO for a final concentration of 10 μm SAHA. 5 µL of 1 mm Boc-Lys(Ac)-AMC (APExBIO Technology LLC, Houston, TX) was added to each reaction to a final concentration of 100 μm. The reactions, at a final volume of 50 µL, were performed at 37 °C for 1 h. Reactions were quenched with 2.5 µl of 200 μm SAHA and incubated at 37 °C for 5 min. Six µL of 50 mg/ml trypsin from porcine pancreas (Sigma Aldrich) was added to the reactions for a final concentration of 5 mg/ml. Reactions were incubated an additional 1 h at 37 °C. Fluorescence was measured with a SPECTRAmax GEMINI XS (Molecular Devices, San Jose, CA) using an excitation wavelength of 355, an emission wavelength of 460 nm, and a cutoff wavelength of 455 nm.

##### Western Blotting

Proteins were separated on polyacrylamide gels and transferred to Amersham Pharmacia Biotech™ Hybond™ 0.2 μm PVDF membranes (GE Healthcare Life Science, Marlborough, MA). Blots were probed with a 1:3000 dilution of rabbit-anti-SIN3A (#ab3479 Abcam, Cambridge, MA) or a 1:5000 dilution of mouse-anti-SIN3B (sc-13145x Santa Cruz Biotechnology, Dallas, TX). Membranes were then probed with a 1:10,000 dilution of IRDye® 680LT Goat-anti-Mouse (LI-COR, Lincoln, NE), IRDye® 800CW Goat-anti-Mouse (LI-COR), or a 1:10,000 dilution of IRDye® 800CW Goat-anti-Rabbit (LI-COR). Images were acquired with an Odyssey® CLx (LI-COR).

##### Sequence Alignments

A pairwise alignment of SIN3A and SIN3B_2 (supplemental Fig. S1) was generated using the EMBOSS-Needle algorithm (30). An alignment of SIN3A (NP_001138829.1), SIN3B_1 (NP_056075.1), SIN3B_2 (NP_001284524.1), and SIN3B_3 (NP_001284526.1) in supplemental Fig. S2 was generated using ETE v3 (31) and ClustalO (32).

## RESULTS

##### SIN3A and SIN3B Interaction Networks Partially Overlap

**Fig. 1:**
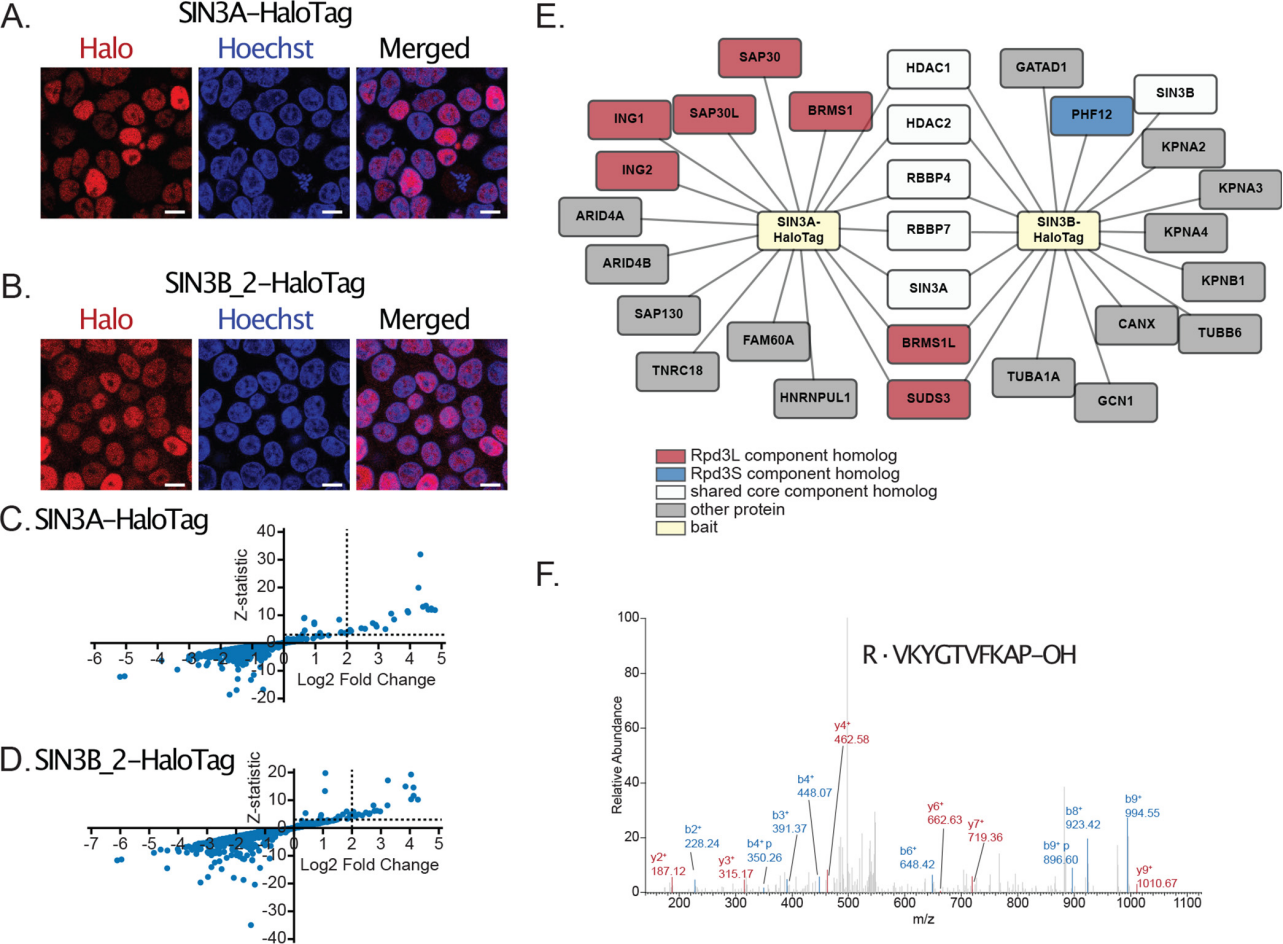
**Analysis of recombinant SIN3A and SIN3B_2 interaction networks.***A*–*B*, Subcellular localization of stably expressed (*A*) SIN3A (NP_001138829.1) and (*B*) SIN3B_2 (NP_001284524.1) in Flp-In™-293 cells. HaloTag® TMRDirect™ Ligand and Hoechst 33258 solution were used to visualize recombinant protein localization (*red*) and nuclei (*blue*), respectively. White bars indicate 10 μm. *C*–*D*, Plots of Z-statistic *versus* log2 fold change for the proteins detected in each APMS analysis of (*C*) SIN3A and (*D*) SIN3B_2 (supplemental Table S2D). Filter values used for enriched protein identification, Z-statistics ≥ 3 and log2 fold change values ≥ 2, are represented as dashed lines. *E*, Network of proteins with at least one isoform enriched by SIN3A (SIN3A-HaloTag) and/or SIN3B isoform 2 (SIN3B_2-HaloTag). Recombinant forms of SIN3A and SIN3B_2 are source nodes (*yellow*). Proteins with homology to Rpd3L-specific components (red), proteins with homology to Rpd3S-specific components (blue), and proteins with homology to proteins found within both Rpd3L and Rpd3S complexes (white) are displayed. Proteins with no clear homology to yeast Sin3 complex components (gray) are also shown. *F*, Spectrum matching a peptide specific to untagged SIN3A that was observed following SIN3A-HaloTag affinity purification.

As an initial measure to characterize properties of human Sin3 complexes, we stably expressed SIN3A (NM_001145357.2, NP_001138829.1) and SIN3B isoform 2 (transcript NM_001297595.1, NP_001284524.1) as fusions with a HaloTag (Fig. 1*A*–1*B*, supplemental Fig. S3). SIN3B isoform 2 (SIN3B_2) was chosen for analysis as it represents the isoform that most closely resembles SIN3A (supplemental Fig. S2) and also has strong support as the principal/main isoform within transcriptome databases (https://gtexportal.org/home/gene/SIN3B) (33). Halo-tagged proteins were purified and interacting proteins were identified via MudPIT MS (Fig. 1*C*–1*D* and supplemental Table S2).

The identification of protein enrichment over negative controls using QSPEC v1.3.5 (28) (Fig. 1*C*–1*D*, supplemental Tables S2D–2E) revealed that SIN3A and SIN3B_2 both captured 18 proteins (Fig. 1*E*). Although SIN3A and SIN3B_2 enriched the same number of proteins, only 7 proteins were enriched in both SIN3A and SIN3B_2 purifications (Fig. 1*E*). Among proteins enriched by both SIN3A and SIN3B_2 were HDAC1/HDAC2 and RBBP4/RBBP7, proteins with homology to the yeast Sin3 core complex components Rpd3 and Ume1, respectively. Using dNSAF values as indicators of protein abundance, HDAC1/HDAC2 and RBBP4/RBBP7 were identified among the most abundant nonbait proteins in both SIN3A and SIN3B_2 enrichments (supplemental Table S2E).

Most identified proteins were uniquely enriched by either SIN3A or SIN3B_2. SIN3A enriched known homologs of Rpd3L-component homologs, including ING1/ING2, SUDS3/BRMS1/BRMS1L, and SAP30/SAP30L. However, SIN3B_2 only enriched a subset of these proteins, including SUDS3/BRMS1L (Fig. 1*E*). Although peptides mapping to ING1/ING2, BRMS1, and SAP30/SAP30L were observed following SIN3B_2 purification, these proteins did not meet criteria for enrichment (supplemental Table S2E).

Unlike Rpd3L component homologs which were enriched by SIN3A or both SIN3A and SIN3B_2, an Rpd3S component homolog, PHF12, displayed different behavior. Though PHF12 was initially identified as an interaction partner of SIN3A (34, 35), SIN3A-purified samples were devoid of peptides mapping to PHF12. This is consistent with previous reports that PHF12 may be a SIN3B-specific interaction partner (18). GATAD1, a known SIN3B and PHF12 interaction partner (17, 36), was also specifically enriched by SIN3B_2 (Fig. 1*E*). Peptides mapping to other known PHF12 and SIN3B interaction partners, including MORF4L1, EMSY, and KDM5A, were observed in SIN3B_2-purified samples. However, SIN3A-purified samples were devoid of peptides mapping to these proteins (supplemental Table S2E).

Other proteins identified in our analysis of the SIN3A interaction network included the known SIN3A interaction partners FAM60A and TNRC18 (37) as well as the common contaminant protein HNRNPUL1 (38). SIN3B interaction partners included proteins involved in the nuclear import of proteins (KPNA2, KPNA3, KPNA4, KPNB1) (39) and common contaminant proteins (CANX, GCN1, TUBA1A, TUBB6) (38).

Interestingly, SIN3A was enriched by SIN3B_2 (Fig. 1*E*, supplemental Tables S2D–2E). Further, a peptide uniquely mapping to untagged SIN3A was observed following SIN3A-HaloTag enrichment (replicate #2), indicating that two copies of SIN3A may be present in some forms of the Sin3 complex (Fig. 1*F*). These data provide evidence for the existence of homooligomeric and heterooligomeric forms of Sin3 complexes in humans.

##### Rpd3L and Rpd3S Component Homologs Define Subgroups within the Human Sin3 Interaction Network

**Fig. 2:**
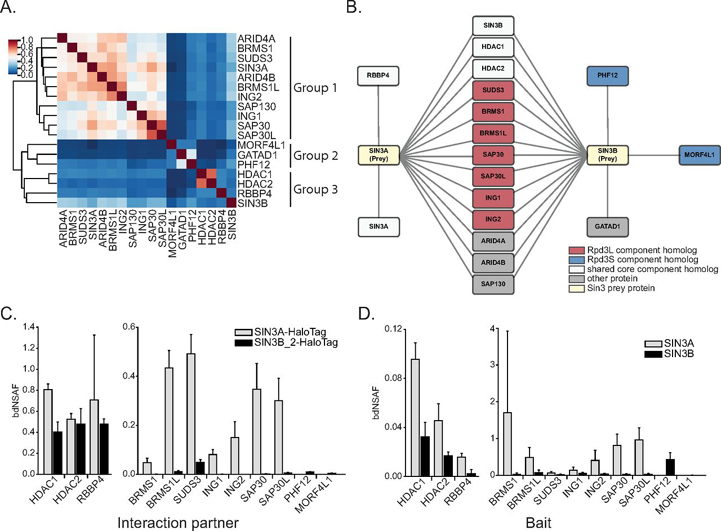
**Construction of the human Sin3 interaction network.***A*, Jaccard similarity indexes calculated using identities of enriched proteins within each bait protein purification were used as input for hierarchical clustering. Clustering was performed using the unweighted pair group method with arithmetic mean algorithm. Clustering of baits reveals 3 groups of proteins. *B*, Network of Halo-tagged bait proteins that enrich SIN3A and/or SIN3B. Halo-tagged bait proteins are target nodes and SIN3A/SIN3B are source nodes (*yellow*). Proteins with homology to Rpd3L-specific components (*red*), proteins with homology to Rpd3S-specific components (blue), and proteins with homology to proteins found within both Rpd3L and Rpd3S complexes (white) are displayed. Proteins with no clear homology to yeast Sin3 complex components (gray) are also shown. *C*, Bait normalized dNSAF (bdNSAF) of Rpd3S and Rpd3L component homologs with peptides observed following SIN3A-HaloTag and/or SIN3B_2-HaloTag affinity purification. *D*, bdNSAF of SIN3A and SIN3B following affinity purification of Rpd3S and Rpd3L component homologs expressed as fusions with HaloTag. *C*–*D*, bdNSAF values were acquired by dividing prey protein dNSAF by bait protein dNSAF.

To validate SIN3A and SIN3B interactions, we expanded our proteomic profiling to include the analysis of bait-purified interaction partners of SIN3A and SIN3B. Sixteen components of the Sin3 interaction network were stably expressed as fusions with HaloTag (supplemental Fig. S4) and enriched proteins were identified following purification of bait proteins (supplemental Fig. S5, supplemental Table S2). We first assessed the degrees to which bait protein interaction networks overlapped by calculating Jaccard similarity indexes for each pair of interaction networks (Fig. 2*A*). Clustering of bait proteins revealed three subgroups within the analyzed bait proteins that have characteristics mapping to different forms of the Sin3 complexes identified in *S. cerevisiae*. The first group of proteins contained human homologs of Rpd3L-specific components, including ING1/ING2, SUDS3/BRMS1/BRMS1L, and SAP30/SAP30L. SIN3A also resided within this cluster, indicating that the interaction networks of SIN3A and Rpd3L component homologs are similar. A second group of proteins consisted of Rpd3S component homologs PHF12 and MORF4L1. A known interaction partner of PHF12 and MORF4L1, GATAD1 (36), also resided within this cluster. The third group of proteins was comprised of proteins with homology to the shared core found within both Rpd3L and Rpd3S complexes, including the proteins HDAC1/HDAC2, RBBP4, and SIN3B_2 (Fig. 2*A*).

We next compared the identities of Halo-tagged bait proteins that enriched SIN3A and/or SIN3B. This analysis of SIN3A and SIN3B interactions showed that HDAC1/HDAC2 enriched both Sin3 paralogs. Although peptides mapping to SIN3B were consistently observed in samples obtained through RBBP4 enrichment, SIN3B did not meet criteria for enrichment (Fig. 2*B*, supplemental Table S2E). This result may represent an artifact associated with the recombinant form of RBBP4 as a large portion of recombinant RBBP4 was observed within the cytoplasm (supplemental Fig. S4L) and recombinant RBBP4 was obtained at low levels (supplemental Table S2E). Thus, the recombinant form of this protein may be partially mis-localized and unstable.

Much like the enrichment of native SUDS3/BRMS1L by SIN3B_2, recombinant SUDS3/BRMS1L enriched native SIN3B (Fig. 2*B*). Unlike native forms of BRMS1, SAP30/SAP30L, and ING1/ING2, which were present in SIN3B-purified samples but failed to meet criteria for enrichment (Fig. 1*E*, supplemental Table S2E), the analysis of the reciprocal interaction consistently revealed an enrichment of SIN3B by these Halo-tagged baits. The analysis of Rpd3L component homologs showed that all of those analyzed were capable of enriching both Sin3 paralogs.

Although Rpd3L components can enrich SIN3B, our analysis of the Sin3 interaction network revealed evidence for paralog-specific interaction behavior (Fig. 2*C*–2*D*). Bait-normalized dNSAF (bdNSAF) values of Rpd3L-specific component homologs were consistently lower following SIN3B_2-HaloTag purification compared with SIN3A-HaloTag enrichment (Fig. 2*C*). Additionally, SIN3A bdNSAF values were consistently higher than those of SIN3B following purification of Halo-tagged ING1/ING2, BRMS1L, and SAP30/SAP30L (Fig. 2*D*, supplemental Table S2E). These results are consistent with ING1/ING2, BRMS1L, and SAP30/SAP30L preferentially interacting with SIN3A.

##### Chemical Cross-linking Mass Spectrometry Identifies Direct Interactions within Human Core Sin3 Complexes

Having profiled interactions within the Sin3 interaction network and identified the proteins that are most highly enriched by SIN3A/SIN3B_2, we next assessed direct interactions within the network and the influence of Sin3 paralog identity on such interactions. To achieve this, we employed chemical cross-linking MS (XL-MS) to identify direct interactions within samples isolated via purification of SIN3A-HaloTag (supplemental Table S3A) or SIN3B_2-HaloTag (supplemental Table S3B) affinity purification.

**Fig. 3:**
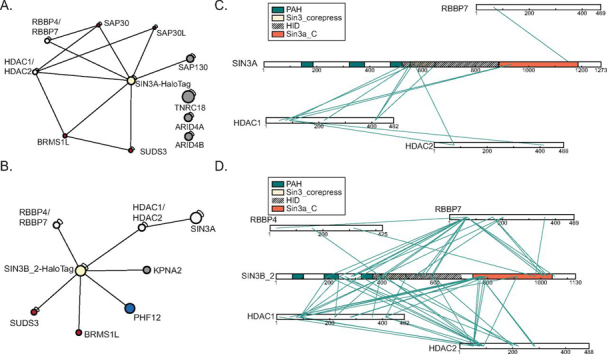
**Chemical cross-linking MS analysis of SIN3A-HaloTag and SIN3B-HaloTag.***A*–*B*, Connectivity maps showing cross-links involving proteins identified in our analysis as enriched by (*A*) SIN3A-HaloTag or (*B*) SIN3B_2-HaloTag (Fig. 1*E*, supplemental Tables S2E, S3). *A–B*, Proteins with homology to Rpd3L-specific components (*red*), proteins with homology to Rpd3S-specific components (*blue*), and proteins with homology to proteins found within both Rpd3L and Rpd3S complexes (*white*) are displayed. Proteins with no clear homology to yeast Sin3 complex components (*gray*) are also shown. *C*–*D*, Maps of identified cross-links between (*C*) SIN3A-HaloTag or (*D*) SIN3B_2-HaloTag and the Sin3 complex components HDAC1/HDAC2 and RBBP4/RBBP7. PAH domains (*green*), Sin3_corepress domains (tan), and Sin3a_C domains (*orange*) are displayed. The experimentally defined SIN3A HID and a highly homologous region within SIN3B are designated by dashed lines.

Components of the shared core of proteins, consisting of HDAC1/HDAC2 and RBBP4/RBBP7, are represented among proteins that cross-linked to both SIN3A and SIN3B_2 (Fig. 3*A*–3*B*). SUDS3/BRMS1L, Rpd3L-component homologs that were enriched by both SIN3A and SIN3B_2 (Fig. 1*E*), were also identified as proteins that cross-linked to both SIN3A and SIN3B_2. Consistent with their enrichment by SIN3A-HaloTag but not SIN3B_2-HaloTag (Fig. 1*E*, supplemental Table S2E), SAP30/SAP30L only cross-linked to SIN3A (Fig. 3*A*, supplemental Table S3A). A cross-link between a PHF12 peptide and a SIN3B_2 peptide was observed (Fig. 3*B*, supplemental Table S3B); however, PHF12 was not identified as a protein cross-linked to SIN3A (Fig. 3*A*, supplemental Table S3A).

##### Highly Conserved Domains within SIN3A and SIN3B Cross-link to Core Complex Components

We next sought to identify domains within SIN3A and SIN3B that mediate protein-protein interactions within Sin3 complexes. The Sin3_corepress (Pfam accession PF08295) domain of SIN3A was highly represented among cross-links that included peptides mapping to HDAC1/HDAC2 (Fig. 3*C*). Consistent with these results, a 327 residue HDAC interaction domain (HID) within mouse SIN3A that is essential for interactions between SIN3A and HDAC2 has been experimentally defined and encompasses the Sin3_corepress domain (40). In addition to the Sin3_corepress domain of SIN3A, we also observed cross-links between HDAC1 and the SIN3A Sin3a_C domain (PF16879) (Fig. 3*C*).

Though a HID within SIN3B has not been experimentally defined, alignment of SIN3A and SIN3B_2 reveals that a region of SIN3B_2 has high homology to the experimentally defined SIN3A HID (supplemental Fig. S2) and encompasses the SIN3B Sin3_corepress domain. HDAC1/HDAC2 cross-linked to a wider range of locations within SIN3B_2 compared with SIN3A; however, clusters of cross-links were observed between HDAC1/HDAC2 and the predicted SIN3B_2 HID (Fig. 3*D*). Like SIN3A, cross-links between HDAC1/HDAC2 and the SIN3B_2 Sin3a_C domain were identified (Fig. 3*D*). These data suggest that HDAC1/HDAC2 interact with at least two annotated domains (Sin3_corepress and Sin3a_C) within both SIN3A and SIN3B_2.

Our analysis also identified a cross-link between RBBP7 and the SIN3A Sin3a_C domain (Fig. 3*C*). RBBP7 cross-linked to a similar location within the SIN3B_2 Sin3a_C domain (Fig. 3*D*). Thus, unlike HDAC1/HDAC2 which cross-linked to the N-terminal portion of the SIN3A and SIN3B_2 Sin3a_C domain, RBBP4 and/or RBBP7 cross-linked to the C-terminal portion of the SIN3A and SIN3B Sin3a_C domains (Fig. 3*C*–3*D*).

##### Rare SIN3B Isoforms Provide Insight into Core Complex Assembly

**Fig. 4:**
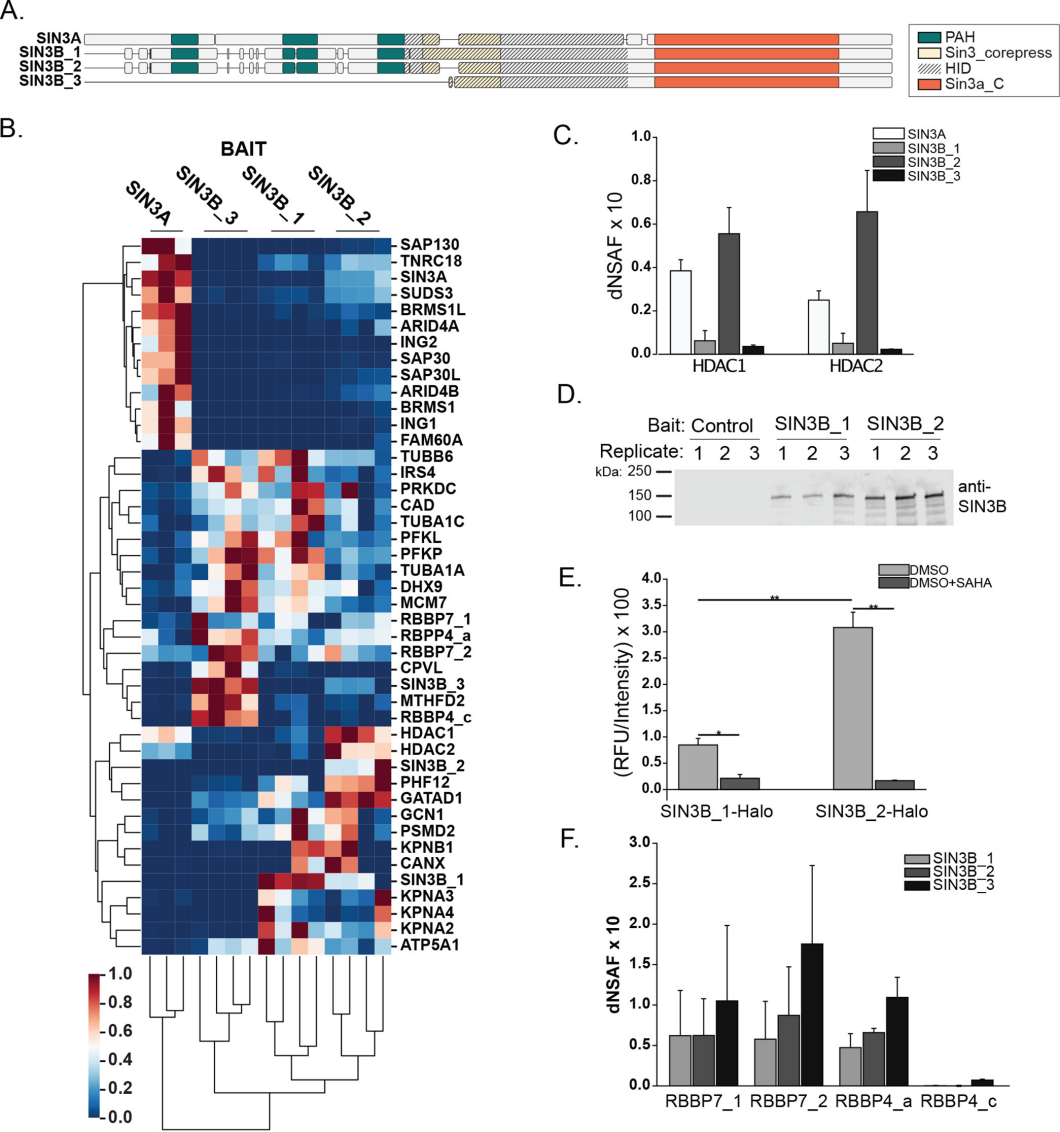
**Assessment of SIN3B domain organization with rare SIN3B isoforms.***A*, Visual alignment of human SIN3A (NP_001138829.1), SIN3B_1 (NP_056075.1), SIN3B_2 (NP_001284524.1), and SIN3B_3 (NP_001284526.1). PAH domains (*green*), Sin3_corepress domains (tan), and Sin3a_C domains (*orange*) are displayed. The experimentally defined SIN3A HID and a highly homologous region within SIN3B are designated by dashed lines. Detailed sequence alignments are provided in supplemental Fig. S2. *B*, Clustered heatmap of normalized dNSAF values for proteins within each bait purification replicate that were enriched by at least one Sin3 bait protein. Proteins were isolated and analyzed from three (SIN3A) or four (all SIN3B isoforms) replicates of cells stably expressing the recombinant Sin3 protein of interest (supplemental Table S4A–S4E). Values were standardized by subtracting the minimum value and dividing by the maximum values for each prey species. Clustering was performed using the unweighted pair group method with arithmetic mean algorithm. Proteins for which multiple isoforms are represented are denoted by isoform identifiers after the protein name. *C*, Average dNSAF values measured for HDAC1 and HDAC2 in SIN3A- (white), SIN3B_1 (light grey), SIN3B_2 (dark grey), and SIN3B_3 (*black*) affinity-purified samples. *Mean* ± *S.D., n* = *3* for SIN3A, and *n = 4* for all SIN3B isoforms (supplemental Table S4E). *D*–*E*, HaloTag (Control), SIN3B_1-HaloTag (SIN3B_1), SIN3B_2-HaloTag (SIN3B_2) were transiently expressed in 293T cells and purified using Magne® HaloTag® Beads. Purified protein was eluted in 100 µL of elution buffer. *D*, Ten µL of eluate from each transfection replicate was loaded onto 4–15% polyacrylamide gels and blots were probed with anti-SIN3B and goat-anti-mouse secondary antibody. *E*, HDAC activity assay of protein complexes purified using SIN3B_1 (SIN3B_1_Halo) and SIN3B_2 (SIN3B_2_Halo) transiently expressed within 293T cells as baits. Reactions were supplemented with DMSO (grey) or DMSO + SAHA (*black*). Relative fluorescence unit (RFU) values for all biological replicates were normalized to recombinant SIN3B protein abundance in purified samples as measured by Western blot (Fig. 3*D*, supplemental Fig. S7, supplemental Table S5). Mean ± S.D., *n* = 3. *: *p* ≤ 0.005, **: *p* ≤ 0.001. *F*, Average dNSAF values measured for RBBP7 and RBBP4 proteoforms in SIN3B_1 (light grey), SIN3B_2 (dark grey), and SIN3B_3 (*black*) affinity-purified samples. Mean ± S.D., *n* = 4 (supplemental Table S4E).

Although isoform 2 likely represents the dominant isoform of SIN3B, Genome Reference Consortium Human Build 38 patch release 13 contains 2 additional annotated isoforms. SIN3B isoform 1 (SIN3B_1, NM_015260.3, NP_056075.1) represents the longest isoform and contains an exon absent within isoform 2 (Fig. 4*A*, supplemental Fig. S2). Isoform 3 (SIN3B_3, NM_001297597.1, NP_001284526.1) results from an alternative start codon and lacks the N-terminal regions found within isoforms 1 and 2 (Fig. 4*A*, supplemental Fig. S2). Because variations present within these SIN3B isoforms reside within regions that our XL-MS analyses identified as domains that at least partially mediate interactions with HDAC1/HDAC2 (Fig. 3*B*, 3*D*), we utilized these SIN3B proteoforms to characterize and validate the identity of the SIN3B HID. SIN3B_1 and SIN3B_3 were expressed with C-terminal HaloTag fusions (supplemental Fig. S6A–S6B) and interaction networks of all Sin3 proteoforms were assessed (Fig. 4*B*, supplemental Fig. S6C–S6F).

Although HDAC1/HDAC2 were enriched by SIN3B_1 and SIN3B_2 (supplemental Table S4E), distributed spectral (dS) counts and dNSAF values of these proteins were consistently lower following SIN3B_1 purification (Fig. 4*B*–4*C*, supplemental Table S4E). HDAC1 also met criteria for enrichment following SIN3B_3 purification; however, HDAC1/HDAC2 were less abundant compared with SIN3B_2-purified samples (Fig. 4*B*–4*C*, supplemental Table S4E). Thus, disruption of the predicted HID by variations present within SIN3B_1 and SIN3B_3 interferes with, but does not completely inhibit, HDAC1/HDAC2 binding. This is consistent with HDAC1/HDAC2 cross-linking to multiple regions within SIN3B_2.

We next sought to investigate what affect the additional exon present within SIN3B_1 had on the catalytic properties of SIN3B complexes. SIN3B_1 and SIN3B_2 with C-terminal HaloTags were transiently expressed in 293T cells (Fig. 4*D*) for subsequent protein isolation and HDAC activity assays. As SIN3B_1 protein levels were consistently lower than those of SIN3B_2 (Fig. 4*D*), activity was normalized to bait protein abundance (Fig. 4*E*, supplemental Fig. S7, supplemental Table S5). The normalized enzymatic activity of SIN3B_1-purified samples was consistently lower than purified complexes containing recombinant SIN3B_2 (Fig. 4*E*). These data provide additional evidence that the experimentally defined HID within SIN3A is also present within SIN3B.

Unlike HDAC1/HDAC2, RBBP4/RBBP7 were observed at comparable levels following purification of SIN3B_1-HaloTag, SIN3B_2-HaloTag, and SIN3B_3-HaloTag (Fig. 4*F*). Thus, the additional exon found within SIN3B_1 does not disrupt association between these proteins and the C-terminal half of SIN3B is sufficient for interactions between SIN3B and RBBP4/RBBP7. This is consistent with RBBP4/RBBP7 cross-linking to the SIN3B_2 Sin3a_C domain and is like previous observations indicating that the deletion of the SIN3A HID does not disrupt interactions between SIN3A and RBBP7 (20).

##### Identification of Cross-links between Sin3 Proteins and Rpd3L/Rpd3S Component Homologs

**Fig. 5:**
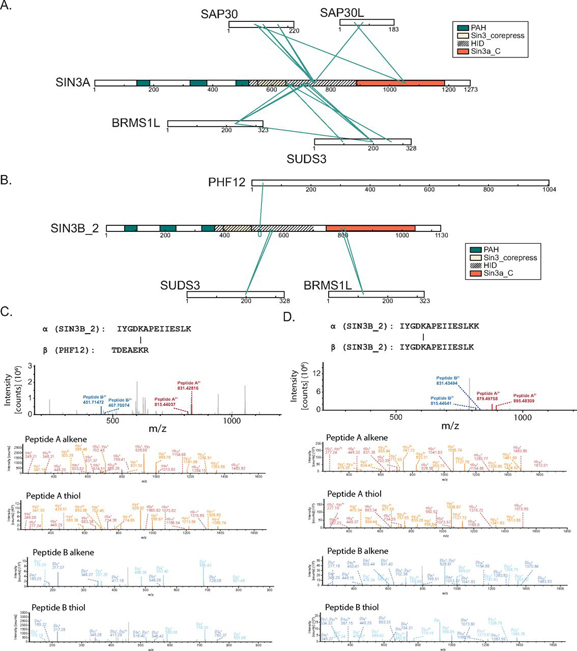
**Identification of interfaces mediating interactions between Rpd3L/Rpd3S component homologs and SIN3A/SIN3B.***A*–*B*, Map of identified cross-links between (*A*) SIN3A-HaloTag or (*B*) SIN3B-HaloTag and proteins with homology to Rpd3L- and Rpd3S-specific component homologs (supplemental Table S3). *A*–*B*, PAH domains (*green*), Sin3_corepress domains (tan), and Sin3a_C domains (*orange*) are displayed. The experimentally defined SIN3A HID and a highly homologous region within SIN3B are designated by dashed lines. *C*, Peptides cross-linked to one another that map to SIN3B_2 and PHF12. MS2 spectrum (top spectrum) and MS3 spectra (bottom 4 spectra) are displayed. *D*, Peptides cross-linked to one another that represent a self-link between separate SIN3B molecules. MS2 spectrum (top spectrum) and MS3 spectra (bottom 4 spectra) are displayed.

SUDS3/BRMS1L were identified as proteins enriched by and directly cross-linked to both SIN3A-HaloTag and SIN3B_2-HaloTag (Fig. 1*E*, 3*A*–3*B*, supplemental Table S2E, supplemental Table S3A–S3B). Cross-links with SUDS3 peptides mapped to the SIN3A HID and the corresponding region within SIN3B_2 (Fig. 5*A*–5*B*). Although BRMS1L also cross-linked to the SIN3A HID (Fig. 5*A*), it cross-linked to the Sin3a_C domain of SIN3B (Fig. 5*B*). In a similar fashion to SUDS3/BRMS1L, SAP30/SAP30L cross-linked to the SIN3A HID. No cross-links were identified between SIN3B_2 and SAP30/SAP30L. Interestingly, no cross-links between the additional Rpd3L component homologs ING1/ING2 and SIN3A/SIN3B_2 were observed (Fig. 3*A*–3*B*, 5*A*–5*B*, supplemental Table S3), despite their enrichment by SIN3A (Fig. 1*E*).

Having acquired data consistent with Rpd3L components directly interacting with SIN3A/SIN3B_2 Sin3_corepress and Sin3a_C domains, we next examined the interaction between SIN3B_2 and the Rpd3S component homolog PHF12. Much like SUDS3, PHF12 cross-linked to the predicted SIN3B_2 HID (Fig. 5*B*–5*C*). Interestingly, a cross-link was observed between peptides within this region from two different SIN3B subunits (Fig. 5*B*, 5*D*). Together, these results support a model in which the conserved HID and Sin3a_C domains are at least partially responsible for the organization of complexes that resemble both Rpd3L- and Rpd3S-complexes and that the HID may also mediate SIN3B dimerization.

##### SIN3A and SIN3B Have Divergent Nuclear Localization Signals

**Fig. 6:**
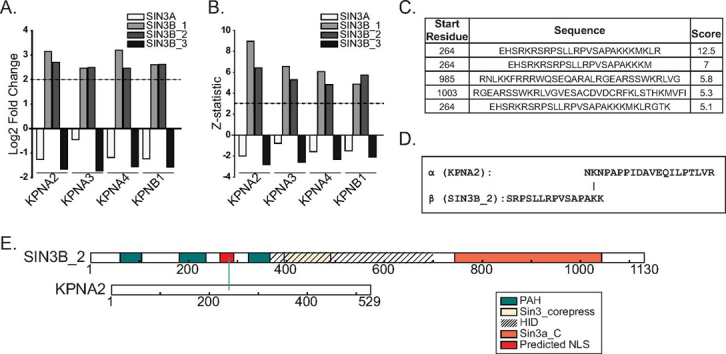
**Assessment of SIN3B interactions with karyophorin proteins.***A*, Log2 fold change and (*B*) Z-statistic values calculated by QSPEC v1.3.5 for KPNA2, KPNA3, KPNA4, and KPNB1 in SIN3A- (white), SIN3B_1- (light grey), SIN3B_2- (dark grey), and SIN3B_3- (*black*) purified samples (supplemental Table S4D). *C*, Potential SIN3B NLS signals identified by cNLS Mapper (41). *D*, Peptides cross-linked to one another that map to KPNA2 and SIN3B_2 (supplemental Table S3B). *E*, Map of identified cross-link between KPNA2 and SIN3B_2. SIN3B_2 PAH domains (*green*), the Sin3_corepress domain (tan), and the Sin3a_C domain (*orange*) are displayed. A region that aligns with the experimentally defined HID region within SIN3A is designated by dashed lines. A predicted NLS with a cNLS mapper score of 12.5 is displayed in red.

Our analysis of the Sin3 interaction network provided insight into the mechanisms responsible for the nuclear import of SIN3B. Although SIN3A, SIN3B_1, and SIN3B_2 were observed within the nucleus, SIN3B_3 was absent within the nucleus (Fig. 1*A*–1*B*, supplemental Fig. S6A–S6B). This localization pattern may indicate that SIN3B_3 lacks a domain required for the nuclear import of this protein or that it is a misfolded protein. In support of SIN3B_3 lacking a nuclear localization signal (NLS), karyophorins KPNA2, KPNA3, KPNA4, and KPNB1 were enriched by SIN3B_1 and SIN3B_2 but not SIN3B_3 (Fig. 4*B*, 6*A*–6*B*, supplemental Table S4E). Notably, SIN3B_1 and SIN3B_2 contain a sequence predicted by cNLS Mapper (41) to be a bipartite NLS (Fig. 6*C*, supplemental Fig. S2). Supporting the prediction of this region as an NLS, we observed a cross-link between KPNA2 and a residue within the top scoring predicted NLS (Fig. 6*D*–6*E*, supplemental Table S3B).

**Fig. 7:**
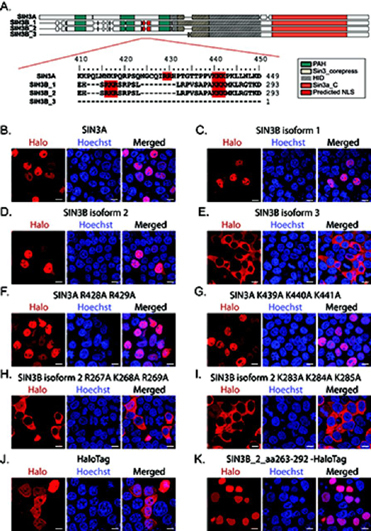
**Characterization of the predicted SIN3B nuclear localization signal.** (*A*, *top panel*) Alignment of the cNLS Mapper (41) predicted bipartite nuclear localization sequence in SIN3B (*red*). PAH domains (green), Sin3_corepress domains (tan), and Sin3a_C domains (orange) are displayed. The experimentally defined SIN3A HID and a highly homologous region within SIN3B are designated by dashed lines. (*A*, *bottom panel*) Residues mutated to test predicted NLS accuracy are highlighted in red. Alignment residue number (*top*) and sequence residue numbers (*right*) are displayed. *B*–*I*, Subcellular localization of transiently expressed WT (*B*–*E*) and mutant (*F*–*I*) forms of Halo-tagged recombinant Sin3 proteoforms in 293T cells. *F*–*I*, Protein names are appended with the identities of mutated residues. *J*–*K*, Subcellular localization of transiently expressed HaloTag (*J*) and a sequence containing the predicted SIN3B NLS, spanning residues 263 to 292 of SIN3B_2, with a C-terminal HaloTag (*K*) in 293T cells. (*B*–*K*) HaloTag® TMRDirect™ Ligand and Hoechst 33258 solution were used to visualize recombinant protein localization (red) and nuclei (blue), respectively. White bars indicate 10 μm.

To test the accuracy of the predicted NLS sequence in SIN3B isoforms, basic residues within the predicted bipartite sequence in SIN3B_2 were mutated to alanine residues. Basic residues found within this region of SIN3A were also mutated (Fig. 7*A*). Open reading frames encoding WT (Fig. 7*B*–7*E*) and mutant (Fig. 7*F*–7*I*) forms of SIN3A and SIN3B proteoforms were transiently expressed in 293T cells as fusions with HaloTag. Mutating residues within either segment of the predicted SIN3B_2 NLS disrupted nuclear localization of the recombinant protein (Fig. 7*H*–7*I*), consistent with this site functioning as a bipartite NLS. Surprisingly, the introduction of mutations to homologous residues in SIN3A did not inhibit the nuclear localization of SIN3A (Fig. 7*F*–7*G*). The observation that KPNA2/KPNA3/KPNA4 and KPNB1 were not enriched by SIN3A purification (Fig. 1*E*, 4*B*, supplemental Table S4E) is consistent with SIN3A and SIN3B_2 undergoing nuclear localization via distinct molecular interactions.

To further validate the identity of the SIN3B NLS, we expressed a sequence containing the predicted NLS (Fig. 6*C* bottom sequence) with a C-terminal HaloTag and examined subcellular localization. The NLS-HaloTag fusion was consistently observed within the nucleus whereas the HaloTag control displayed diffuse localization (Fig. 7*J*–7*K*). These data identify an NLS and importin-α interaction domain that is present within SIN3B_2.

## DISCUSSION

The existence of protein paralogs within the Sin3 interaction network likely results in a compositionally and functionally heterogeneous population of Sin3 complexes. This diversity presents a challenge as we seek to identify beneficial and off-target effects associated with the application of HDAC inhibitors in clinical settings. Before gaining an adequate understanding of Sin3 complex function, we must first define interactions and modularity within the complexes. Through a comparative analysis of SIN3A and SIN3B, we highlight the influence of paralog switching on complex composition and identify unique attributes of these proteins.

##### Inference of Complex Modularity from Protein Interactions

The single *S. cerevisiae* Sin3 protein is partitioned into 2 distinct protein complexes, known as Rpd3S and Rpd3L (5, 6). Common to both complexes is a core group of proteins consisting of Sin3, Rpd3, and Ume1, which share homology with human SIN3A/SIN3B, HDAC1/HDAC2, and RBBP4/RBBP7, respectively. Though the *S. cerevisiae* complexes share a core of components, they have distinct subunits that fine-tune complex function (5, 6). There is evidence that distinct forms of the Sin3 complex exist in humans and that these complexes can be defined based on subunit homology to yeast Sin3 complex components (17, 36, 42). To adequately characterize the Sin3 interaction network, we performed a comparative analysis of Sin3 paralogs, homologs of *S. cerevisiae* Rpd3L and Rpd3S complex components, and other known Sin3 interaction network components.

Our assessment of the Sin3 proteins shows that interaction networks of SIN3A and SIN3B_2 partially overlap. Among proteins enriched by both paralogs were HDAC1/HDAC2 and RBBP4/RBBP7 (Fig. 1*E*). Using chemical cross-linking MS, we show that HDAC1/HDAC2 and RBBP4/RBBP7 likely directly interact with both SIN3A and SIN3B (Fig. 3*A*–3*B*). Thus, complexes constructed upon both SIN3A and SIN3B possess a shared core set of subunits that resembles the Rpd3 core complex.

A complex containing SIN3B, PHF12, MORF4L1, GATAD1, KDM5A, and EMSY has been previously described in humans (36). As PHF12 and MORF4L1 are homologs of Rpd3S components, it is plausible that this complex represents a structure that is partially homologous to the Rpd3S complex. Our data confirm previous findings and show that SIN3B interacts with Rpd3S component homologs (Fig. 1*E*, 2*B*, 5*B*–5*C*). Though SIN3B_2 did not enrich EMSY and KDM5A, peptides mapping to these proteins were present in SIN3B_2-purified samples. Additionally, these proteins were enriched by GATAD1 and PHF12 (supplemental Table S2E). Future assays should characterize the nature of interactions between SIN3B and EMSY/KDM5A and determine if the integration of these components into complexes requires the presence of GATAD1 and/or PHF12.

In contrast to SIN3B, we found no evidence that SIN3A interacts with PHF12 or MORF4L1. Rather, the interaction network of SIN3A closely resembled those of Rpd3L component homologs (Fig. 2*A*) and enrichment of these proteins by SIN3A was observed. These interactions were confirmed with the reciprocal enrichment of native SIN3A by recombinant Rpd3L-specific component homologs (Fig. 2*D*). Together, these data offer strong support for SIN3A being a component of protein modules resembling Rpd3L complexes. Though SIN3A likely exists in such a Rpd3L-like module, Rpd3L-specific component homologs, namely ING1/ING2 and SAP30/SAP30L, also consistently enriched SIN3B. However, the degree of enrichment was much less than that of SIN3A (Fig. 2*D*). Peptides mapping to BRMS1/BRMS1L/SUDS3, ING1/ING2, and SAP30/SAP30L were also observed following SIN3B_2 purification but only BRMS1L/SUDS3 met criteria for enrichment. These observations suggest that either these components preferentially interact with SIN3A or that interactions with SIN3B are indirect and require linker molecules. Regardless of the nature of protein interactions, our results show that SIN3B does not specifically integrate into Rpd3S-like complexes.

During our assessment of the Sin3 interaction network, we obtained data that indicate Sin3 complexes contain multiple copies of the Sin3 scaffold proteins. We found direct evidence for the existence of complexes that contain multiple copies of either SIN3A (Fig. 1*F*) or SIN3B (Fig. 5*B*, 5*D*). In addition to data that indicates homooligomers exist, heterooligomeric forms of the complex likely exist as SIN3B_2 enriched SIN3A (Fig. 1*E*). The presence of multiple copies of Sin3 proteins within a single complex may indicate that Sin3 complexes are composed of separate modules, each of which contain a separate Sin3 protein.

**Fig. 8:**
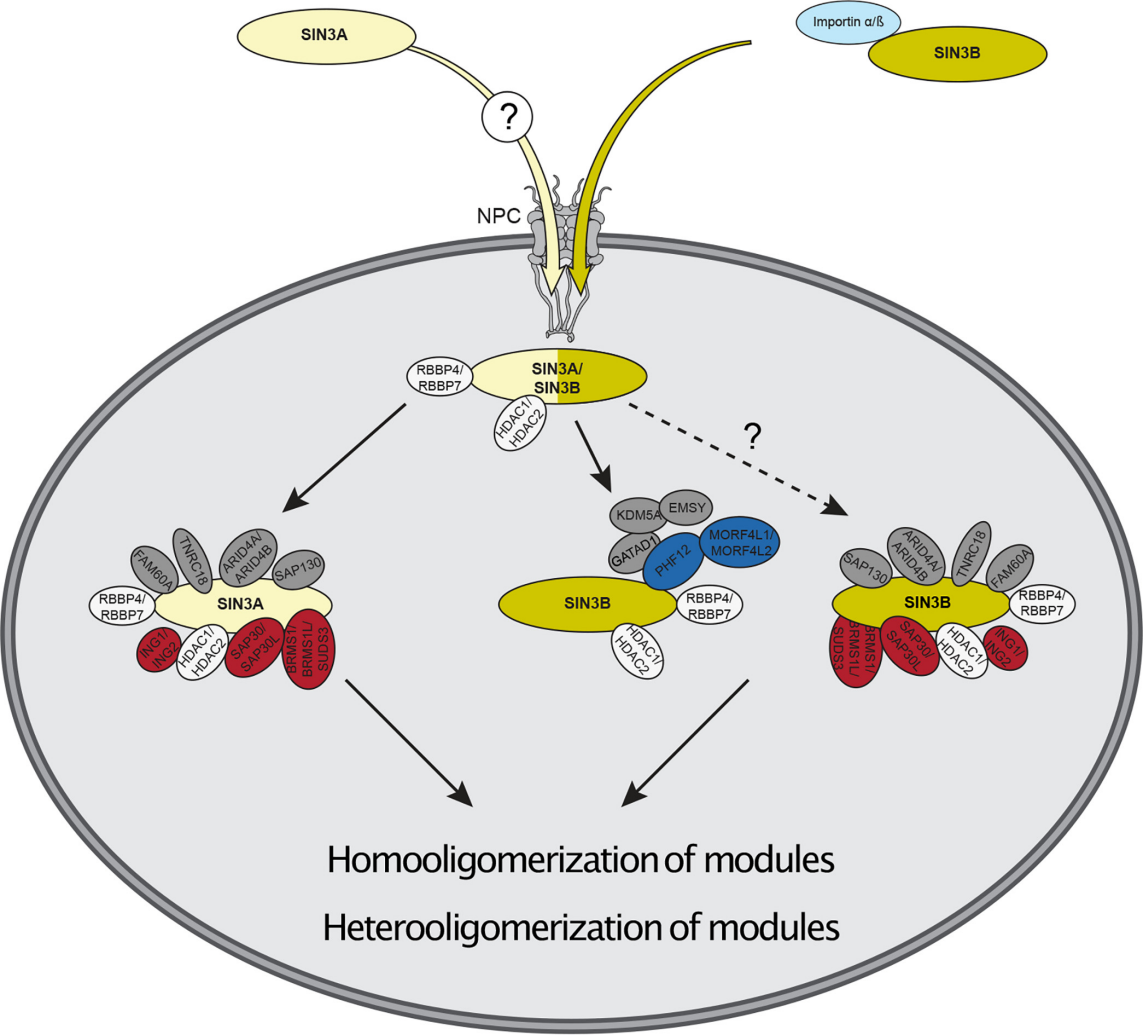
**Model of the human SIN3A and SIN3B interactions defined in this study.** SIN3A and SIN3B have divergent nuclear localization signals that mediate their nuclear import. The SIN3B NLS serves as recognition signal for importin (light blue) and mediates nuclear import. PHF12 and known PHF12 interaction partners likely represent SIN3B-specific interaction partners. Homooligomerization and heterooligomerization of Sin3 modules may occur. NPC: nuclear pore complex. Rpd3L component homologs (*red*), Rpd3S component homologs (dark blue), Rdp3L/Rpd3S shared component homologs (white), and other known Sin3 interaction partners (gray) are displayed.

A model based upon complex modularity and oligomerization may explain the weak interactions we observed between SIN3B and many Rpd3L component homologs. As SIN3A directly interacts with SAP30/SAP30L and SIN3B_2 enriches SIN3A, the incorporation of SAP30/SAP30L into SIN3B_2-containing complexes may require heterooligomerization of modules and the presence of SIN3A as a linking molecule (Fig. 8). Though we found evidence that 2 subunits of SIN3B directly interact (Fig. 5*B*), previous findings suggest that yeast Sds3 is essential for Sin3 complex integrity (43) and that mammalian BRMS1 (44) and SUDS3 (14, 44) are capable of forming dimers. As SUDS3 and BRMS1L were enriched by both SIN3A and SIN3B_2, it is possible that these proteins mediate the formation of, or stabilize, SIN3A-SIN3B_2 heterooligomeric complexes. Future assays should aim to characterize the nature of Sin3 module dimerization and what roles BRMS1, BRMS1L, and SUDS3 play in this process.

Interestingly, interactions between Rpd3L and Rpd3S component homologs were not observed in our assay conditions. However, results obtained by others indicate that PHF12 may be capable of interacting, either directly or indirectly, with Rpd3L component homologs (17). Thus, it is likely that chimeric complexes that consist of both Rpd3L and Rpd3S component homologs (Fig. 8) exist within humans under certain conditions. Weak associations between Rpd3L- and Rpd3S-component homologs in these assays may be explained by a model that accounts for heterodimerization of SIN3A and SIN3B modules (Fig. 8).

##### XL-MS Highlights the Roles That Conserved Regions within Sin3 Proteins Play in Complex Construction

SIN3A and SIN3B possess several domains that have retained a high degree of homology (Fig. 4*A*). Three PAH domains reside in the N-terminal halves of these proteins and are proposed to mediate interactions between Sin3 proteins and several transcription factors (45). C-terminal to the PAH domains are the Sin3_corepress and Sin3a_C domains that share 96 and 75% sequence similarity, respectively, between SIN3A and SIN3B. Such high degrees of similarity suggest that these domains serve conserved roles within both paralogs. The experimentally defined HID of SIN3A encompasses the Sin3_corepress and is needed for interactions between SIN3A and HDAC1/HDAC2 (40, 44, 46).

Although our proteomic profiling provides insight into the identities of components within interaction networks, it does not discriminate between direct or indirect interactions nor does it identify protein domains that mediate interactions. To gain insight into direct protein-protein interactions within Sin3 complexes, we examined interaction interfaces as determined by XL-MS. The shared group of core complex proteins, consisting of HDAC1/HDAC2 and RBBP4/RBBP7, cross-linked to both SIN3A and SIN3B_2. Direct interactions between the SIN3A HID and these proteins are consistent with previous descriptions (20). Although a HID has not been experimentally defined within SIN3B, an alignment of mouse SIN3A and SIN3B protein sequences shows that residues 388 to 651 of SIN3B_2 have approximately 88% sequence similarity to residues 545 to 808 in SIN3A.

HDAC1/HDAC2 and RBBP4/RBBP7 cross-linked to several regions of SIN3B_2, though the predicted HID is highly represented among cross-links. We demonstrate that isoform 1, which contains a 32-residue sequence not found within isoform 2, only weakly enriches HDAC1/HDAC2 and has a decreased activity (Fig. 4*C*–4*E*, supplemental Table S2). Thus, the addition of this exon does not completely abolish interactions with HDAC1/HDAC2 but does diminish both HDAC1/HDAC2 binding and the complex's catalytic activity. This is consistent with SIN3A and SIN3B sharing conserved domains that are required for the basal function associated with the complexes.

Within humans, HDAC1/HDAC2 and RBBP4/RBBP7 exist in multiple protein complexes and cross-links have been identified between HDAC1 and RBBP4 through the analysis of the NuRD complex (47). Interestingly, we identified no cross-links between HDAC1/HDAC2 and RBBP4/RBBP7 in SIN3A-HaloTag and SIN3B_2-HaloTag complexes. Although these results do not rule out direct interactions between these components within the core Sin3 complex, disruption of SIN3B interactions with HDAC1/HDAC2 via the inclusion of an additional exon within the HID did not disrupt RBBP4/RBBP7 integration into the complexes. These data indicate that RBBP4/RBBP7 incorporation into Sin3 complexes is not dependent upon the presence of HDAC1/HDAC2.

In addition to HDAC1/HDAC2, we mapped cross-links between Sin3 proteins and Rpd3L and Rpd3S component homologs. It has been previously shown that the interaction between SUDS3 and SIN3A requires an intact HID (14). In accordance with previous descriptions (20), we observed cross-links between SUDS3 and the SIN3A HID (Fig. 5*A*). In addition to SIN3A, we also observed cross-links between SUDS3 and the predicted SIN3B_2 HID. Interestingly, BRMS1L, a potential functional homolog of SUDS3, cross-linked to the Sin3a_C domain of SIN3B_2 (Fig. 5*B*). Thus, binding of these subunits to Sin3 scaffolding subunits may occur via unique domains or interactions between these proteins and SIN3B may require multiple domains within SIN3B.

It has also been shown that removal of the predicted SIN3B HID disrupts interactions between PHF12 and SIN3B (18). Consistent with this observation, we map an interaction between the N terminus of PHF12 and the predicted HID of SIN3B_2 (Fig. 5*B*–5*C*). Taken together, these results suggest that the organizing role of the HID region is conserved between SIN3A and SIN3B_2. Additionally, the presence of cross-links between Sin3 protein HIDs and both Rpd3L and Rpd3S component homologs suggests that the HID may serve as a central scaffolding region for Rpd3L-like and Rpd3S-like modules.

##### The Composition of the SIN3B Interaction Network Provides Insight into Mechanisms Responsible for SIN3B Nuclear Import

In addition to providing clues regarding Sin3 complex modularity, our profiling of the SIN3B interaction network also identified interactions between SIN3B and proteins that mediate the nuclear import of proteins. Importin α/importin β heterodimers mediate the translocation of proteins into the nucleus via the nuclear pore complex (48). SIN3B_1/SIN3B_2 enriched several importin subunits, including KPNA2/KPNA3/KPNA4 and KPNB1, whereas the cytoplasmic SIN3B_3 failed to enrich these proteins (Fig, 6*A*–6*B*, supplemental Table S4E). XL-MS assays revealed a direct interaction between a predicted NLS within SIN3B_2 and KPNA2. Notably, this predicted NLS is absent within SIN3B_3. A sequence containing the predicted SIN3B NLS, expressed as a fusion with HaloTag, localized within the nucleus (Fig. 7*K*). Thus, our data describe an NLS within SIN3B that is required for the nuclear import of SIN3B.

Interestingly, a region within SIN3A that aligns with the SIN3B NLS does not appear to influence the nuclear localization of SIN3A. A recent report showed that a truncated form of SIN3A mislocalized within the cytoplasm (49). As this SIN3A variant resulted from a nonsense mutation at residue 944, the C terminus of SIN3A is likely critical for nuclear import. These data are consistent with SIN3A and SIN3B undergoing nuclear translocation via unique molecular interactions.

## CONCLUSIONS

The assessment of interactions within the Sin3 interaction network provides insight into the shared and unique properties of human Sin3 scaffolding proteins. Our findings highlight the influence that Sin3 paralog switching has on protein complex composition and outline the need for future studies that further delineate the unique functions of the distinct classes of Sin3 complexes. As many proteins within the Sin3 interaction network exist in equilibrium with paralogous protein, future studies should consider additional heterogeneity within the population of Sin3 complexes that is introduced by nonSin3 protein paralogs.

## Data Availability

Original data underlying this manuscript can be accessed from the Stowers Original Data Repository at http://www.stowers.org/research/publications/libpb-1445. Mass spectrometry data was uploaded to MassIVE repository (http://massive.ucsd.edu) using identifiers listed in supplemental Table S1B.
