# Supplementary material for: Differential Complex Formation via Paralogs in the Human Sin3 Protein Interaction Network
Source: Mol Cell Proteomics. 2020 Nov 25;19(9):1468–84. doi: 10.1074/mcp.RA120.002078 (PMC8143632; doi:10.1074/mcp.RA120.002078)
Supplement: Supplementary file 1 [file mmc1.zip › mmc1/159999_0_supp_506292_qb255s.pdf]

Supplemental Materials for:

Differential Complex Formation via Paralogs in the Human Sin3 Protein Interaction Network

Mark K. Adams[1], Charles A.S. Banks[1], Janet L. Thornton[1], Cassandra G. Kempf[1], Ying Zhang[1], Sayem Miah[1], Yan Hao[1], Mihaela E. Sardiu[1], Maxime Killer[1,2], Gaye L. Hattem[1], Alexis Murray[1], Maria L. Katt[1], Laurence Florens[1], Michael P. Washburn[1,3,*]

[1] Stowers Institute for Medical Research, Kansas City, MO 64110

[2] Current address: Centre for Structural Systems Biology (CSSB), DESY and European Molecular Biology Laboratory Hamburg, Hamburg, Germany

[3] Department of Pathology & Laboratory Medicine, University of Kansas Medical Center, Kansas City, KS 66160

[*]Correspondence: mpw@stowers.org

Supplemental Methods:

Expression construct preparation-

HaloTag-ARID4A_pcDNA5/FRT- The Kazusa DNA Research Institute (KDRI) clone #FHC12701, which codes for ARID4A isoform 3 in the parental vector pFN21A, was modified to code for isoform 1 (NP_002883) as follows. The codon optimized synthetic sequence shown in **Figure S8A** was digested with KpnI and cloned into the KpnI site in FHC12701 (replacing a sequence at the 3' end of the isoform 3 ORF) to generate ARID4A isoform 1 in pFN21A. ARID4A isoform 1 was then excised using SgfI and PmeI, and subcloned between the PacI and PmeI sites in the vector Halo pcDNA5/FRT PacI PmeI (which uses the CMV promoter) that has been previously described (supplemental reference 1).

HaloTag-ARID4B_pcDNA5/FRT- The KDRI clone #FHC01194 was modified by site directed mutagenesis to add a stop codon immediately upstream of the PmeI site. This sequence, coding for ARID4B isoform 1 (NP_001193723) was then excised using SgfI and PmeI, and subcloned between the PacI and PmeI sites in the vector Halo pcDNA5/FRT PacI PmeI that has been previously described (supplemental reference 1).

HaloTag-BRMS1_pcDNA5/FRT- Human placental total RNA (Clontech, Palo Alto, CA) was used to prepare cDNA using the iScript cDNA synthesis kit (Bio-Rad, Hercules, CA). The BRMS1 primers listed in **Table S1A** were used to amplify a sequence coding for BRMS1 isoform 1 (NP_056214) from cDNA. This sequence was digested with SgfI and PmeI. The fragment was cloned between the PacI and PmeI sites in the vector CMVd2 Halo pcDNA5/FRT PacI PmeI (which uses the CMVd2 promoter) that has been previously described (supplemental reference 2).

HaloTag-BRMS1L_pcDNA5/FRT- The BRMS1L primers listed in **Table S1A** were used to amplify a sequence coding for BRMS1L (NP_115728) from cDNA (prepared as described for HaloTag-BRMS1_pcDNA5/FRT). The PCR product was digested with SgfI and PmeI and then inserted between the PacI and PmeI sites in the vector CMVd2 Halo pcDNA5/FRT PacI PmeI that has been previously described (supplemental reference 2).

HaloTag-GATAD1_pcDNA5/FRT- A gBlock® (Integrated DNA Technologies, Coralville, IA) containing a codon optimized sequence encoding GATAD1 (NP_066990), provided in **Figure S8B**, was digested with SgfI and PmeI and cloned into PacI and PmeI sites in the vector CMVd2 Halo pcDNA5/FRT PacI PmeI that has been previously described (supplemental reference 2).

HDAC1-HaloTag_pcDNA5/FRT- HDAC1 in pFN21A was amplified with primers described in **Table S1A**. The amplicon was digested with SgfI and PmeI and cloned into SgfI and Eco53KI sites of pFC16K to add an in-frame C-terminal HaloTag. The CMVd2 promoter, HDAC1 ORF (encoding NP_004955), and HaloTag in pFC16K were then amplified with primers described in **Table S1A**. The amplicon was subsequently digested and cloned into MluI and NotI sites of pcDNA5/FRT Mammalian Expression Vector.

HDAC2-HaloTag_pcDNA5/FRT- A codon optimized sequence encoding HDAC2, provided in **Figure S8C**, was synthesized commercially in the vector pUCIDT-AMP (Integrated DNA Technologies), excised by digestion with SgfI and PmeI, and then subcloned between the SgfI and PmeI sites of pFN21A. This sequence was amplified with primers described in **Table S1A**. The resulting amplicon was digested with SgfI and PmeI then cloned into SgfI and Eco53KI sites of pFC14A. The CMV promoter, HDAC2 ORF (encoding NP_001518), and HaloTag in pFC14A were then amplified with primers

described in **Table S1A**. The amplicon was subsequently digested and cloned into MluI and NotI sites of pcDNA5/FRT Mammalian Expression Vector.

HaloTag-ING1_pcDNA5/FRT- The ING1 primers listed in **Table S1A** were used to amplify a sequence coding for ING1 isoform A (NP_937862, also known as ING1b) from cDNA prepared as described above for BRMS1. This sequence was digested with SgfI and PmeI and then cloned between the PacI and PmeI sites in the vector CMVd2 Halo pcDNA5/FRT PacI PmeI that has been previously described (supplemental reference 2).

HaloTag-ING2_pcDNA5/FRT- The ING2 primers listed in **Table S1A** were used to amplify a sequence coding for ING2 isoform 1 (NP_001555) from cDNA prepared as described above for BRMS1. This sequence was digested with SgfI and PmeI for cloning between the PacI and PmeI sites in the vector CMVd2 Halo pcDNA5/FRT PacI PmeI that has been previously described (supplemental reference 2).

MORF4L1-HaloTag_pcDNA5/FRT- A gBlock® (Integrated DNA Technologies) with a codon optimized sequence encoding MORF4L1 (NP_006782), provided in **Figure S8D**, was digested with SgfI and PmeI and cloned into SgfI and PmeI sites of pFN21A vector. The insert was then amplified with primers described in **Table S1A**. The amplicon was digested with SgfI and cloned into SgfI and Eco53KI sites of pFC14A to add an in-frame C-terminal HaloTag. The CMV promoter, MORF4L1 ORF, and HaloTag in pFC14A were excised with MluI and NotI then cloned into MluI and NotI sites of pcDNA5/FRT.

HaloTag-PHF12_pcDNA5/FRT- PHF12 in pcDNA3.1 (encoding NP_001028733) was amplified with primers described in **Table S1A**. The amplicon was digested with SgfI and cloned into SgfI and PmeI of pFN21A, then digested and cloned into PacI and PmeI sites in the vector CMVd2 Halo pcDNA5/FRT PacI PmeI that has been previously described (supplemental reference 2).

HaloTag-RBBP4_pcDNA5/FRT- KDRI clone #FHC10501 (coding for RBBP4 isoform a, NP_005601) was used as a template for a PCR reaction using the RBBP4 primers described in **Table S1A**. The PCR product was digested with SgfI and PmeI and cloned between the PacI and PmeI sites in the vector CMVd2 Halo pcDNA5/FRT PacI PmeI that has been previously described (supplemental reference 2).

HaloTag-SAP130_pcDNA5/FRT- The KDRI clone #FHC01565, which codes for SAP130 isoform a (NP_001139400), was modified by site directed mutagenesis to insert a stop codon upstream of the PmeI site. The SAP130 insert was then excised with SgfI and PmeI and inserted between the PacI and PmeI sites in the vector Halo pcDNA5/FRT PacI PmeI that has been previously described (supplemental reference 1).

SIN3A-HaloTag_pcDNA5/FRT- The V109A point mutation present in KDRI clone #FHC11647 was corrected using targeted mutagenesis. SIN3A was then excised with SgfI and PmeI then into cloned into SgfI and Eco53KI sites of pFC14A to add a C-terminal HaloTag®. The CMV promoter and SIN3A (encoding NP_001138829) with an in-frame C-terminal HaloTag® were excised and cloned into MluI and NotI sites of pcDNA5/FRT Mammalian Expression Vector.

SIN3B_1-HaloTag_pcDNA5/FRT- The MiniGene™ (Integrated DNA Technologies) described in **Fig. S8E** was digested with NsiI and BsiWI and cloned into these restriction sites of KDRI clone #FHC01991. This construct was then digested with SgfI and BsiWI then cloned into SgfI and BsiWI sites of the below described SIN3B_2 in pFC14A to create SIN3B_1 with a C-terminal HaloTag®. The CMV promoter and SIN3B_1 (encoding NP_056075) with an in-frame C-terminal HaloTag® were excised and cloned into MluI and NotI sites of pcDNA5/FRT Mammalian Expression Vector.

SIN3B_2-HaloTag_pcDNA5/FRT- KDRI clone #FHC01991 was digested with SgfI and PmeI, then cloned into SgfI and Eco53KI sites of pFC14A to add a C-terminal HaloTag®. The CMV promoter and SIN3B_2 (encoding NP_001284524) with an in-frame C-terminal HaloTag® were excised and cloned into MluI and NotI sites of pcDNA5/FRT Mammalian Expression Vector.

SIN3B_3-HaloTag_pcDNA5/FRT- A gBlock® (Integrated DNA Technologies), described in **Fig. S8F** was digested with SgfI and BsiWI then cloned into the above described SIN3B_2-pFC14A. The CMV promoter and SIN3B_3 (encoding NP_001284526) with an in-frame C-terminal HaloTag® was excised and cloned into MluI and NotI sites of pcDNA5/FRT Mammalian Expression Vector.

SIN3A-HaloTag_R428A/R429A_pFC14A, SIN3A-HaloTag_K439A/K440A/K441A_pFC14A, SIN3B_2-HaloTag_R267A/K268A/R269A_pFC14A, and SIN3B_2-HaloTag_K283A/K284A/K285A_pFC14A- Site-directed mutagenesis was performed to introduce point mutations in SIN3A or SIN3B_2 in pFC14A.

SIN3B_2_aa263-292_pFC14A- A gBlock® (Integrated DNA Technologies) spanning residues 263 to 292 of SIN3B_2, described in **Fig. S8G**, was digested with SgfI and PmeI then cloned into SgfI and Eco53KI sites of pFC14A.

Supplemental References:

1. Banks, C.A.S., Lee, Z.T., Boanca, G., Lakshminarasimhan, M, Groppe, B.D., Wen, Z., Hattem, G.L., Seidel, C.W., Florens, L., and Washburn, M.P. (2014) Controlling for gene expression changes in transcription factor protein networks. *Mol. Cell. Proteomics* 13, 1510-1522
2. Banks, C.A.S., Boanca, G., Lee, Z.T., Florens, L., Washburn, M.P. (2015) Proteins interacting with cloning scars: a source of false positive protein-protein interactions. *Sci. Rep.* 5, 8530

Supplemental Tables:

Table S1 Related to Figures 1, 2, 3, 4, 5, 6, and 7. Oligonucleotides and Data Availability.

Table S2 Related to Figures 1 and 2. Identification, label-free quantitation, and statistical analysis of proteins detected in APMS analyses of Sin3 interaction network components

Table S3 Related to Figures 3, 5, and 6. Identification of DSSO-crosslinked peptides in APMS analyses of Sin3 interaction network components

Table S4 Related to Figures 4, and 6. Identification, label-free quantitation, and statistical analysis of proteins detected in SIN3A and SIN3B isoform enriched samples

Table S5 Related to Figure 4. Calculation of SIN3B_1 and SIN3B_2 HDAC activity

Supplemental Figures:

Supplementary Figure 1 Related to Figure 1, 3, and 5. Pairwise sequence alignment of SIN3A (NP_001138829) and SIN3B isoform 2 (NP_001284524) generated using EMBOSS-Needle (30). PAH domains (light gray), Sin3_corepress (dark gray) and Sin3a_C (black) domains are displayed.

Supplementary Figure 2 Related to Figures 4, 6, and 7. Protein alignment of SIN3A and SIN3B isoforms generated with ClustalO v1.2.4 (32). A sequence within SIN3B_1 and SIN3B_2 that possesses a cNLS Mapper score of 12.5 as a bipartite NLS is highlighted in gray. A sequence within SIN3A that possesses a cNLS Mapper score of 4.0 as a bipartite NLS is also highlighted in gray. Residues within human SIN3A that are homologous to the mouse SIN3A HID (40) are highlighted in black.

Supplementary Figure 3 Related to Figure 1. Analysis of relative expression levels of native and recombinant Sin3 proteins. (A-B) 45 µg of cell extract from Flp-In™-293 Cells or Flp-In™-293 Cells stably expressing (A) SIN3A-HaloTag or (B) SIN3B_2-HaloTag was loaded per lane. Proteins were separated on a 10% polyacrylamide gel and probed with (A) anti-SIN3A followed by IRDye® 800CW Goat-anti-Rabbit or (B) anti-SIN3B followed by IRDye® 680LT Goat-anti-Mouse.

Supplementary Figure 4 Related to Figure 2. Subcellular localization patterns of recombinant components of the Sin3 interaction network. Halo-tagged components of the Sin3 interaction network were stably expressed within Flp-In™-293 Cells. HaloTag TMRDirect Ligand and Hoechst 33258 solution were used to visualize recombinant protein localization (red) and nuclei (blue), respectively. White bars indicate 10 µm.

Supplementary Figure 5 Related to Figure 2. Identification of proteins enriched in APMS analyses of components of the Sin3 interaction network. Plots of Z-statistic vs log2 fold change for the proteins detected in each APMS analysis of components of the Sin3 interaction network (**Table S2D**). Z-statistic values of 3 or greater and log2 fold change values of 2 or greater were used to identify enriched proteins and are designated as dashed lines on plots.

Supplementary Figure 6 Related to Figures 4 and 6. Subcellular localization patterns of recombinant SIN3A and SIN3B isoforms and identification of proteins enriched in APMS analyses of Sin3 proteins. (A) SIN3B_1-HaloTag and (B) SIN3B_3-HaloTag were stably expressed within Flp-In™-293 Cells. HaloTag TMRDirect Ligand and Hoechst 33258 solution were used to visualize recombinant protein localization (red) and nuclei (blue), respectively. White bars indicate 10 μm. (C-F) Plots of Z-statistic vs log2 fold change for the proteins detected in each APMS analysis of (C) SIN3A-HaloTag, (D) SIN3B_1-HaloTag, (E) SIN3B_2-HaloTag, and (F) SIN3B_3-HaloTag (**Table S4D**). Z-statistic values of 3 or greater and log2 fold change values of 2 or greater were used to identify enriched proteins and are designated as dashed lines on plots.

Supplementary Figure 7 Related to Figure 4D-E. Image of Western blot shown in **Fig. 4D** with intensity values displayed. Transfections and assays were performed in triplicate.

Supplementary Figure 8 Related to Figures 2, 4, 6, and 7. (A) ARID4A isoform 1 (NP_002883) insert MiniGene™ (Integrated DNA Technologies) in pIDTSmart. KpnI sites are underlined. (B) The gBlock® (Integrated DNA Technologies) used to generate a codon-optimized form of GATAD1 (NP_066990). SgfI and PmeI sites are underlined. (C) Codon-optimized HDAC2 (encoding NP_001518) open reading frame. (D) The gBlock® used to generate a codon-optimized form of MORF4L1 (NP_006782). SgfI and PmeI sites are underlined (E) The MiniGene™ sequence used to create SIN3B isoform 1 (NP_056075). NsiI and BsiWI sites are underlined. (F) The gBlock® used to create SIN3B isoform 3 (NP_001284526). SgfI and BsiWI recognition sequences are underlined. (G) The gBlock® used to generate a sequence containing the predicted SIN3B NLS, spanning residues 263 to 292, with an in-frame with a C-terminal HaloTag. SgfI and PmeI sites are underlined.

Supplementary Figure 1 | Adams et al.

Needle Pairwise Sequence Alignment

**Protein**	**Accession**
SIN3A:	NP_001138829
SIN3B isoform 2:	NP_001284524

```
Program: needle
        -datafile EBLOSUM62
        -gapopen 10.0
        -gapextend 0.5
        -endopen 10.0
        -endextend 0.5
        -Matrix: EBLOSUM62
        -Gap_penalty: 10.0
        -Extend_penalty: 0.5

Length: 1295
Identity:     626/1295 (48.3%)
Similarity:   812/1295 (62.7%)
Gaps:         187/1295 (14.4%)
Score: 3068.0

NP_001138829      1 MKRRLDDQESPVYAAQQRRIPGSTEAFPHQHRVLAPAPPVYEAVSETMQS     50
                                                                       
NP_001284524      1 --------------------------------------------------      0

NP_001138829     51 ATGIQYSVTPSYQVSAMPQS------SGSHGPAIAAVHSSHHHPTAVQPH     94
                                    |..:      ||:.|||...:..:....:.    
NP_001284524      1 ----------------MAHAGGGSGGSGAGGPAGRGLSGARWGRSG----     30

NP_001138829     95 GGQVVQSHAHPAPPVAPVQGQQQFQRLKVEDALSYLDQVKLQFGSQPQVY    144
                          |..|...||            .|||||:||||||::|||.|..|
NP_001284524     31 ------SAGHEKLPV------------HVEDALTYLDQVKIRFGSDPATY     62

NP_001138829    145 NDFLDIMKEFKSQSIDTPGVISRVSQLFKGHPDLIMGFNTFLPPGYKIEV    194
                    |.||:||||||||||||||||.||||||..|||||:|||.|||.||:|::
NP_001284524     63 NGFLEIMKEFKSQSIDTPGVIRRVSQLFHEHPDLIVGFNAFLPLGYRIDI    112

NP_001138829    195 QTNDMVNVTTPGQVHQIPTHGIQPQPQPPPQHPSQPSAQSAPAPAQPAPQ    244
                    ..|..:|:.:|                    ..||.::.:....|:...|
NP_001284524    113 PKNGKLNIQSP--------------------LTSQENSHNHGDGAEDFKQ    142

NP_001138829    245 PPPAKVSKPSQLQAHTPASQQTPPLPPYASPRSPPVQPHTPVTISLGTAP    294
                    ..|.|..||           |.|                           
NP_001284524    143 QVPYKEDKP-----------QVP---------------------------    154

NP_001138829    295 SLQNNQPVEFNHAINYVNKIKNRFQGQPDIYKAFLEILHTYQKEQRNAKE    344
                     |:::. ||||:||:||||||.||...|:||::||||||||||||.|.: 
NP_001284524    155 -LESDS-VEFNNAISYVNKIKTRFLDHPEIYRSFLEILHTYQKEQLNTR-    201

NP_001138829    345 AGGNYTPALTEQEVYAQVARLFKNQEDLLSEFGQFLPDANSSVLLSKTTA    394
                      |.....::|:||:.:||.||:.|||||||||||||:|..|:.......
NP_001284524    202 --GRPFRGMSEEEVFTEVANLFRGQEDLLSEFGQFLPEAKRSLFTGNGPC    249

NP_001138829    395 EKVDSVRNDHGGTVKKPQLNNKPQRPSQNGCQIRRHPTGTTPPVKKKPKL    444
                    |.....:|:|.   |.|:.:.|..|||    .:|    ..:.|.|||.||
NP_001284524    250 EMHSVQKNEHD---KTPEHSRKRSRPS----LLR----PVSAPAKKKMKL    288

NP_001138829    445 LNLKDSSMADASKHGGGTESLFFDKVRKALRSAEAYENFLRCLVIFNQEV    494
                    ...||.|:|...|:|...|..||||||:.|:|.|.|||||||:.:||||:
NP_001284524    289 RGTKDLSIAAVGKYGTLQEFSFFDKVRRVLKSQEVYENFLRCIALFNQEL    338
```

Supplementary Figure 1 | Adams et al.

```
NP_001138829    495 ISRAELVQLVSPFLGKFPELFNWFKNFLGYKESVHLETYP--KERATEGI    542
                    :|.:||:||||||||||||||..||:|||.||    |...|  .:|:.:||
NP_001284524    339 VSGSELLQLVSPFLGKFPELFAQFKSFLGVKE---LSFAPPMSDRSGDGI    385

NP_001138829    543 AMEIDYASCKRLGSSYRALPKSYQQPKCTGRTPLCKEVLNDTWVSFPSWS    592
                    :.|||||||:|||||||||:||||||:|||.:||||||||||||||||||
NP_001284524    386 SREIDYASCKRIGSSYRALPKTYQQPKCSGRTAICKEVLNDTWVSFPSWS    435

NP_001138829    593 EDSTFVSSKKTQYEEHIYRCEDERFELDVVLETNLATIRVLEAIQKKLSR    642
                    |||||||||||.|||.::||||||||||||||||||||||||::||||||
NP_001284524    436 EDSTFVSSKKTPYEEQLHRCEDERFELDVVLETNLATIRVLESVQKKLSR    485

NP_001138829    643 LSAEEQAKFRLDNTLGGTSEVIHRKALQRIYADKAADIIDGLRKNPSIAV    692
                    ::.|:|.|||||::||||||||.|:|:.|||.|||.:||:.:|||..||
NP_001284524    486 MAPEDQEKFRLDDSLGGTSEVIQRRAIYRIYGDKAPEIIESLKKNPVTAV    535

NP_001138829    693 PIVLKRLKMKEEEWREAQRGFNKVWREQNEKYYLKSLDHQGINFKQNDTK    742
                    |:||||||.|||||||||:||||:||||.||.||||||||.:||||||||
NP_001284524    536 PVVLKRLKAKEEEWREAQQGFNKIWREQYEKAYLKSLDHQAVNFKQNDTK    585

NP_001138829    743 VLRSKSLLNEIESIYDERQEQATEENAGVPVGPHLSLAYEDKQILEDAAA    792
                    .|||||||||||:|||.|||.:|..:.....|||...|||:|||||||||
NP_001284524    586 ALRSKSLLNEIESVYDEHQEQHSEGRSAPSSEPHLIFVYEDRQILEDAAA    635

NP_001138829    793 LIIHHVKRQTGIQKEDKYKIKQIMHHFIPDLLFAQRGDLSDVEEEEEEEM    842
                    ||.::||||..|||||:..|.|::|.|:|.|.|:|:.||...||..:|:.
NP_001284524    636 LISYYVKRQPAIQKEDQGTIHQLLHQFVPSLFFSQQLDLGASEESADEDR    685

NP_001138829    843 D-----VDEATGAVKKHNGVGGSPPKSKLLFSNTAAQKL---------RG    878
                    |     ..:.:...|...|...|||:.|..|.:..|.:.         :.
NP_001284524    686 DSPQGQTTDPSERKKPAPGPHSSPPEEKGAFGDAPATEQPPLPPPAPHKP    735

NP_001138829    879 MDEVYNLFYVNNNWYIFMRLHQILCLRLLRICSQAERQIEEENREREWER    928
                    :|:||:||:.|||||.|:||||.||.|||:|..||::|:.|...|:|.|:
NP_001284524    736 LDDVYSLFFANNNWYFFLRLHQTLCSRLLKIYRQAQKQLLEYRTEKEREK    785

NP_001138829    929 EVLGIKRDKSDSPAIQLRLKEPMDVDVEDYYPAFLDMVRSLLDGNIDSSQ    978
                    .:...:|:|...||::||||:|.:|::|:|||||||||||||:|:||.:|
NP_001284524    786 LLCEGRREKGSDPAMELRLKQPSEVELEEYYPAFLDMVRSLLEGSIDPTQ    835

NP_001138829    979 YEDSLREMFTIHAYIAFTMDKLIQSIVRQLQHIVSDEICVQVTDLYLAEN   1028
                    |||:||||||||||:.||||||:|:|.|||.|:|||::|::|.:|||.|.
NP_001284524    836 YEDTLREMFTIHAYVGFTMDKLVQNIARQLHHLVSDDVCLKVVELYLNEK    885

NP_001138829   1029 NNGATGGQLNTQNSRSLLESTYQRKAEQLMSDENCFKLMFIQSQGQVQLT   1078
                    ..||.||.|:::..|:..|::||.|||:.|:||||||:||:|:|.:||.:|
NP_001284524    886 KRGAAGGNLSSRCVRAARETSYQWKAERCMADENCFKVMFLQRKGQVIMT    935

NP_001138829   1079 IELLDTEEENSDDPVEAERWSDYVERYMNSDTTSPELREHLAQKPVFLPR   1128
                    ||||||||..::||||.:..:.|||:|:.::..|....|....|||||.|
NP_001284524    936 IELLDTEEAQTEDPVEVQHLARYVEQYVGTEGASSSPTEGFLLKPVFLQR    985

NP_001138829   1129 NLRRIRKCQRGREQQEKEGKEGNSKKTMENVDSLDKLECRFKLNSYKMVY   1178
                    ||::.|:..:..:.:...|:..:|.|.:..|:|...::||||||:::|||:
NP_001284524    986 NLKKFRRRWQSEQARALRGEARSSWKRLVGVESACDVDCRFKLSTHKMVF   1035

NP_001138829   1179 VIKSEDYMYRRTALLRAHQSHERVSKRLHQRFQAWVDKWTKEHVPREMAA   1228
                    ::.||||||..|.||.|....|..|.||.|:.|..:|.:::|..|.|:
NP_001284524   1036 IVNSEDYMYRRGTLCRAKQVQPLVLLRHHQHFEEWHSRWLEDNVTVEAAS   1085

NP_001138829   1229 ETSKWLMGEGLEGLVPCTTTCDTETLHFVSINKYRVKYGTVFKAP   1273
                    ....|||||..|.:|||.|.|:|..:|.:.:.:|||:|.....:|
NP_001284524   1086 LVQDWLMGEEDEDMVPCKTLCETVHVHGLPVTRYRVQYSRRPASP   1130
```

Supplementary Figure 2 | Adams et al.

```
SIN3A:              NP_001138829.1
SIN3B isoform 1:    NP_056075.1
SIN3B isoform 2:    NP_001284524.1
SIN3B isoform 3:    NP_001284526.1
```

```
NP_001138829.1      MKRRLDDQESPVYAAQQRRIPGSTEAFPHQHRVLAPAPPVYEAVSETMQSATGIQYSVTP      60
NP_001284526.1      ------------------------------------------------------------      0
NP_056075.1         ------------------------------------------------------------      0
NP_001284524.1      ------------------------------------------------------------      0

NP_001138829.1      SYQVSAMPQSSGSHGPAIAAVHSSHHHPTAVQPHGGQVVQSHAHPAPPVAPVQGQQQFQR      120
NP_001284526.1      ------------------------------------------------------------      0
NP_056075.1         ------MAHAGGGSGGSG-----------AGGPAGRGLSGARW----G-RSGSAGHEKLP      38
NP_001284524.1      ------MAHAGGGSGGSG-----------AGGPAGRGLSGARW----G-RSGSAGHEKLP      38

NP_001138829.1      LKVEDALSYLDQVKLQFGSQPQVYNDFLDIMKEFKSQSIDTPGVISRVSQLFKGHPDLIM      180
NP_001284526.1      ------------------------------------------------------------      0
NP_056075.1         VHVEDALTYLDQVKIRFGSDPATYNGFLEIMKEFKSQSIDTPGVIRRVSQLFHEHPDLIV      98
NP_001284524.1      VHVEDALTYLDQVKIRFGSDPATYNGFLEIMKEFKSQSIDTPGVIRRVSQLFHEHPDLIV      98

NP_001138829.1      GFNTFLPPGYKIEVQTNDMVNVTTPGQVHQIPT-HGIQPQPQPPPQHPSQPSAQSAPAPA      239
NP_001284526.1      ------------------------------------------------------------      0
NP_056075.1         GFNAFLPLGYRIDIPKNGKLNIQSPLTSQENSHNHGD-----------------GA----      137
NP_001284524.1      GFNAFLPLGYRIDIPKNGKLNIQSPLTSQENSHNHGD-----------------GA----      137

NP_001138829.1      QPAPQPPPAKVSKPSQLQAHTPASQQTPPLPPYASPRSPPVQPHTPVTISLGTAPSLQNN      299
NP_001284526.1      ------------------------------------------------------------      0
NP_056075.1         --------------EDFKQQ----------VPYKED-----KPQ-----------VPLES      157
NP_001284524.1      --------------EDFKQQ----------VPYKED-----KPQ-----------VPLES      157

NP_001138829.1      QPVEFNHAINYVNKIKNRFQGQPDIYKAFLEILHTYQKEQRNAKEAGGNYTPALTEQEVY      359
NP_001284526.1      ------------------------------------------------------------      0
NP_056075.1         DSVEFNNAISYVNKIKTRFLDHPEIYRSFLEILHTYQKEQLNTR---GRPFRGMSEEEVF      214
NP_001284524.1      DSVEFNNAISYVNKIKTRFLDHPEIYRSFLEILHTYQKEQLNTR---GRPFRGMSEEEVF      214

NP_001138829.1      AQVARLFKNQEDLLSEFGQFLPDANSSVLLSKTTAEKVDSVRNDHGGTVKKPQLNNKPQR      419
NP_001284526.1      ------------------------------------------------------------      0
NP_056075.1         TEVANLFRGQEDLLSEFGQFLPEAKRSLFTGNGPCEMHSVQKNEHDKTPEH---SRKRSR      271
NP_001284524.1      TEVANLFRGQEDLLSEFGQFLPEAKRSLFTGNGPCEMHSVQKNEHDKTPEH---SRKRSR      271

NP_001138829.1      PSQNGCQIRRHPTGTTPPVKKKPKLLNLKDSSMADASKHGGGTESLFFDKVRKALRSAEA      479
NP_001284526.1      ------------------------------------------------------------      0
NP_056075.1         PSL--------LRPVSAPAKKKMKLRGTKDLSIAAVGKYGTLQEFSFFDKVRRVLKSQEV      323
NP_001284524.1      PSL--------LRPVSAPAKKKMKLRGTKDLSIAAVGKYGTLQEFSFFDKVRRVLKSQEV      323

NP_001138829.1      YENFLRCLVIFNQEVISRAELVQLVSPFLGKFPELFNWFKNFLGYKESVHLETYPKERAT      539
NP_001284526.1      ------------------------------------------------------------      0
NP_056075.1         YENFLRCIALFNQELVSGSELLQLVSPFLGKFPELFAQFKSFLGVKELSFA-PPMSDRSG      382
NP_001284524.1      YENFLRCIALFNQELVSGSELLQLVSPFLGKFPELFAQFKSFLGVKELSFA-PPMSDRSG      382

NP_001138829.1      EGIAMEIDYASCKRLGSSYRALPKSYQQPKCTGRTPLCKE--------------------      579
NP_001284526.1      --------------------------------------------------------MQRH      4
NP_056075.1         DGISREIDYASCKRIGSSYRALPKTYQQPKCSGRTAICKELDHWTLLQGSWTDDYCMSKF      442
NP_001284524.1      DGISREIDYASCKRIGSSYRALPKTYQQPKCSGRTAICKE--------------------      422

NP_001138829.1      ------------VLNDTWVSFPSWSEDSTFVSSKKTQYEEHIYRCEDERFELDVVLETNL      627
NP_001284526.1      S----RHFLLVQVLNDTWVSFPSWSEDSTFVSSKKTPYEEQLHRCEDERFELDVVLETNL      60
NP_056075.1         KNTCWIPGYSAGVLNDTWVSFPSWSEDSTFVSSKKTPYEEQLHRCEDERFELDVVLETNL      502
NP_001284524.1      ------------VLNDTWVSFPSWSEDSTFVSSKKTPYEEQLHRCEDERFELDVVLETNL      470
                                ************************ ***:::*****************
```

Supplementary Figure 2 | Adams et al.

```
NP_001138829.1      ATIRVLEAIQKKLSRLSAEEQAKFRLDNTLGGTSEVIHRKALQRIYADKAADIIDGLRKN      687
NP_001284526.1      ATIRVLESVQKKLSRMAPEDQEKFRLDDSLGGTSEVIQRRAIYRIYGDKAPEIIESLKKN      120
NP_056075.1         ATIRVLESVQKKLSRMAPEDQEKFRLDDSLGGTSEVIQRRAIYRIYGDKAPEIIESLKKN      562
NP_001284524.1      ATIRVLESVQKKLSRMAPEDQEKFRLDDSLGGTSEVIQRRAIYRIYGDKAPEIIESLKKN      530
                    *******::******:: *:* *****::********:*:*: ***.*** :**:.*:**

NP_001138829.1      PSIAVPIVLKRLKMKEEEWREAQRGFNKVWREQNEKYYLKSLDHQGINFKQNDTKVLRSK      747
NP_001284526.1      PVTAVPVVLKRLKAKEEEWREAQQGFNKIWREQYEKAYLKSLDHQAVNFKQNDTKALRSK      180
NP_056075.1         PVTAVPVVLKRLKAKEEEWREAQQGFNKIWREQYEKAYLKSLDHQAVNFKQNDTKALRSK      622
NP_001284524.1      PVTAVPVVLKRLKAKEEEWREAQQGFNKIWREQYEKAYLKSLDHQAVNFKQNDTKALRSK      590
                    *  ***:****** *********:****:**** ** ********.:********.****

NP_001138829.1      SLLNEIESIYDERQEQATEENAGVPVGPHLSLAYEDKQILEDAAALIIHHVKRQTGIQKE      807
NP_001284526.1      SLLNEIESVYDEHQEQHSEGRSAPSSEPHLIFVYEDRQILEDAAALISYYVKRQPAIQKE      240
NP_056075.1         SLLNEIESVYDEHQEQHSEGRSAPSSEPHLIFVYEDRQILEDAAALISYYVKRQPAIQKE      682
NP_001284524.1      SLLNEIESVYDEHQEQHSEGRSAPSSEPHLIFVYEDRQILEDAAALISYYVKRQPAIQKE      650
                    ********:***:*** :* .:.    *** :.***:********** ::**** .****

NP_001138829.1      DKYKIKQIMHHFIPDLLFAQRGDLSDVEEEEEEEMDVDEAT-----GAVKKHNGVGGSPP      862
NP_001284526.1      DQGTIHQLLHQFVPSLFFSQQLDLGASEESADEDRDSPQGQTTDPSERKKPAPGPHSSPP      300
NP_056075.1         DQGTIHQLLHQFVPSLFFSQQLDLGASEESADEDRDSPQGQTTDPSERKKPAPGPHSSPP      742
NP_001284524.1      DQGTIHQLLHQFVPSLFFSQQLDLGASEESADEDRDSPQGQTTDPSERKKPAPGPHSSPP      710
                    *: .*:*::*:*:*.*:*:*: **.  **. :*: *  :.        *   *  .***

NP_001138829.1      KSKLLFSNTAA---------QKLRGMDEVYNLFYVNNNWYIFMRLHQILCLRLLRICSQA      913
NP_001284526.1      EEKGAFGDAPATEQPPLPPPAPHKPLDDVYSLFFANNNWYFFLRLHQTLCSRLLKIYRQA      360
NP_056075.1         EEKGAFGDAPATEQPPLPPPAPHKPLDDVYSLFFANNNWYFFLRLHQTLCSRLLKIYRQA      802
NP_001284524.1      EEKGAFGDAPATEQPPLPPPAPHKPLDDVYSLFFANNNWYFFLRLHQTLCSRLLKIYRQA      770
                    :.*  *.:: *            : :*:**.**:.*****:*:**** ** ***:*  **

NP_001138829.1      ERQIEEENREREWEREVLGIKRDKSDSPAIQLRLKEPMDVDVEDYYPAFLDMVRSLLDGN      973
NP_001284526.1      QKQLLEYRTEKEREKLLCEGRREKGSDPAMELRLKQPSEVELEEYYPAFLDMVRSLLEGS      420
NP_056075.1         QKQLLEYRTEKEREKLLCEGRREKGSDPAMELRLKQPSEVELEEYYPAFLDMVRSLLEGS      862
NP_001284524.1      QKQLLEYRTEKEREKLLCEGRREKGSDPAMELRLKQPSEVELEEYYPAFLDMVRSLLEGS      830
                    ::*: * . *:* *: :   :*:*...**::****:* :*::*:*************:*.

NP_001138829.1      IDSSQYEDSLREMFTIHAYIAFTMDKLIQSIVRQLQHIVSDEICVQVTDLYLAENNNGAT      1033
NP_001284526.1      IDPTQYEDTLREMFTIHAYVGFTMDKLVQNIARQLHHLVSDDVCLKVVELYLNEKKRGAA      480
NP_056075.1         IDPTQYEDTLREMFTIHAYVGFTMDKLVQNIARQLHHLVSDDVCLKVVELYLNEKKRGAA      922
NP_001284524.1      IDPTQYEDTLREMFTIHAYVGFTMDKLVQNIARQLHHLVSDDVCLKVVELYLNEKKRGAA      890
                    ** :****:**********:.******:*.*.***:*:***::*::*.:*** *::.**:

NP_001138829.1      GGQLNTQNSRSLLESTYQRKAEQLMSDENCFKLMFIQSQGQVQLTIELLDTEEENSDDPV      1093
NP_001284526.1      GGNLSSRCVRAARETSYQWKAERCMADENCFKVMFLQRKGQVIMTIELLDTEEAQTEDPV      540
NP_056075.1         GGNLSSRCVRAARETSYQWKAERCMADENCFKVMFLQRKGQVIMTIELLDTEEAQTEDPV      982
NP_001284524.1      GGNLSSRCVRAARETSYQWKAERCMADENCFKVMFLQRKGQVIMTIELLDTEEAQTEDPV      950
                    **:*.::  *:  *::** ***: *:******:**:*:* :*** :********* :::***

NP_001138829.1      EAERWSDYVERYMNSDTTSPELREHLAQKPVFLPRNLRRIRKCQRGREQQEKEGKEGNSK      1153
NP_001284526.1      EVQHLARYVEQYVGTEGASSSPTEGFLLKPVFLQRNLKKFRRRWQSEQARALRGEARSSW      600
NP_056075.1         EVQHLARYVEQYVGTEGASSSPTEGFLLKPVFLQRNLKKFRRRWQSEQARALRGEARSSW      1042
NP_001284524.1      EVQHLARYVEQYVGTEGASSSPTEGFLLKPVFLQRNLKKFRRRWQSEQARALRGEARSSW      1010
                    *.:: : ***:*:.:: :* .  * :  ***** ***:::*:  :..: :  .*:  .*

NP_001138829.1      KTMENVDSLDKLECRFKLNSYKMVYVIKSEDYMYRRTALLRAHQSHERVSKRLHQRFQAW      1213
NP_001284526.1      KRLVGVESACDVDCRFKLSTHKMVFIVNSEDYMYRRGTLCRAKQVQPLVLLRHHQHFEEW      660
NP_056075.1         KRLVGVESACDVDCRFKLSTHKMVFIVNSEDYMYRRGTLCRAKQVQPLVLLRHHQHFEEW      1102
NP_001284524.1      KRLVGVESACDVDCRFKLSTHKMVFIVNSEDYMYRRGTLCRAKQVQPLVLLRHHQHFEEW      1070
                    * : .*:*  .::*****.::***::::******** :* **:* :  *  * **:*: *

NP_001138829.1      VDKWTKEHVPREMAAETSKWLMGEGLEGLVPCTTTCDTETLHFVSINKYRVKYGTVFKAP      1273
NP_001284526.1      HSRWLEDNVTVEAASLVQDWLMGEEDEDMVPCKTLCETVHVHGLPVTRYRVQYSRRPASP      720
NP_056075.1         HSRWLEDNVTVEAASLVQDWLMGEEDEDMVPCKTLCETVHVHGLPVTRYRVQYSRRPASP      1162
NP_001284524.1      HSRWLEDNVTVEAASLVQDWLMGEEDEDMVPCKTLCETVHVHGLPVTRYRVQYSRRPASP      1130
                     .:* :::*  * *: ...*****  *.:***.* *:*  :* : :.:***:*.    :*
```

Supplementary Figure 3 | Adams et al.

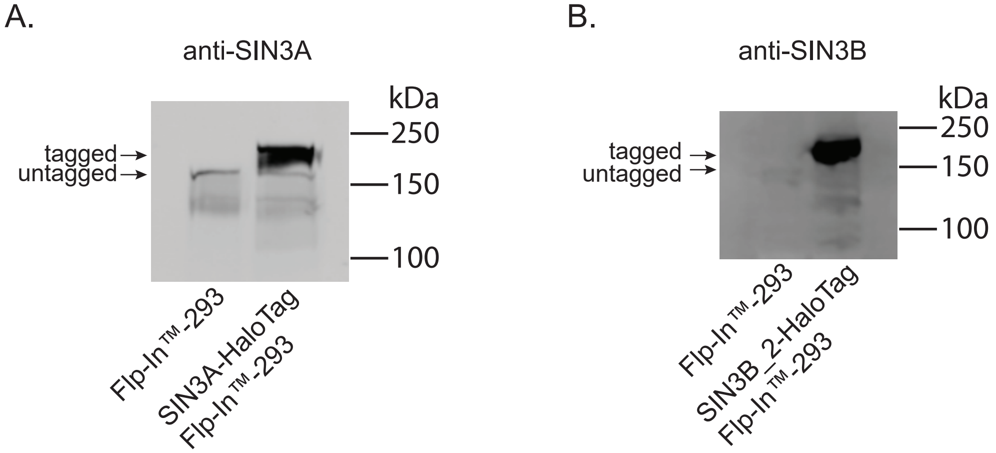

Supplementary Figure 4 | Adams et al.

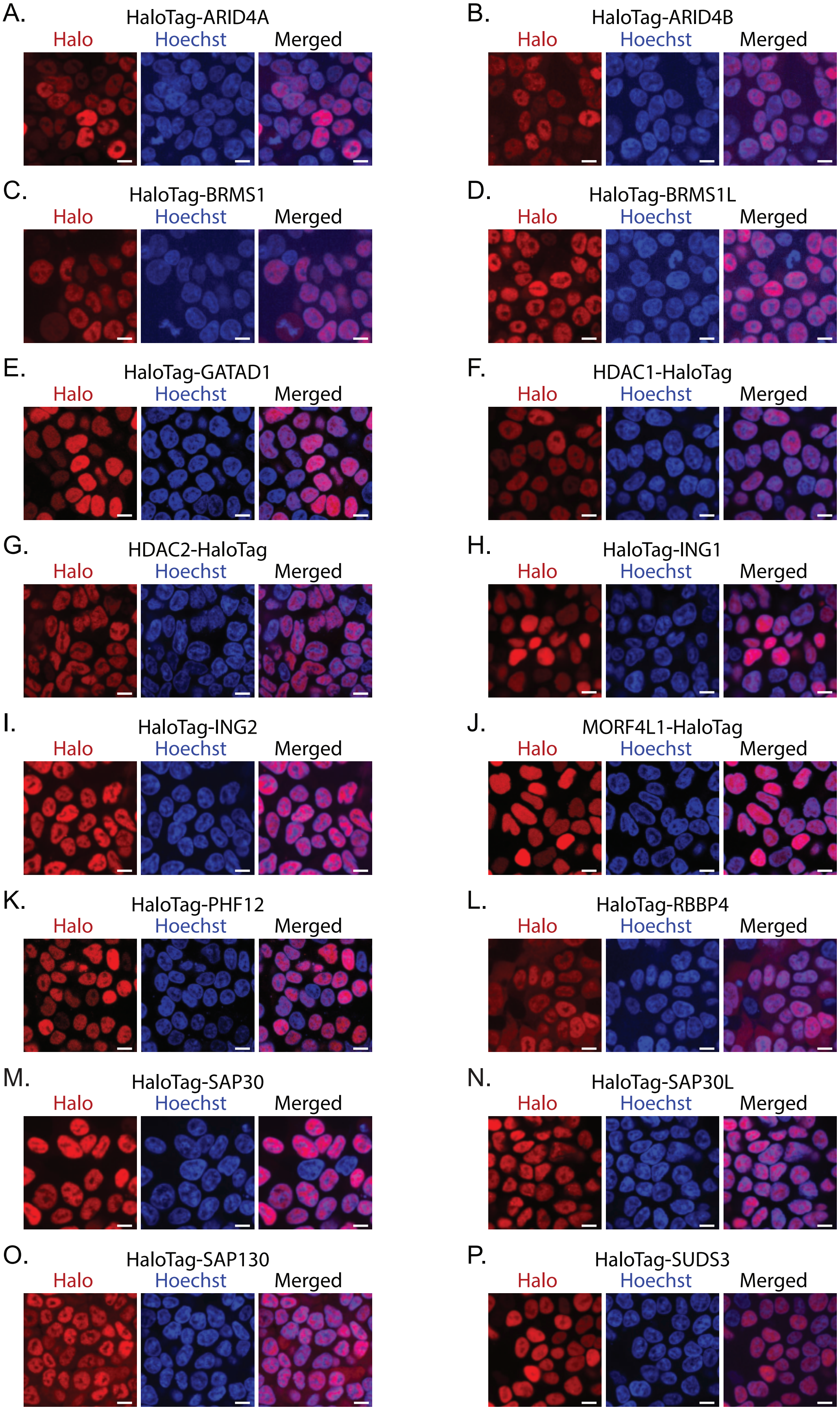

Supplementary Figure 5 | Adams et al.

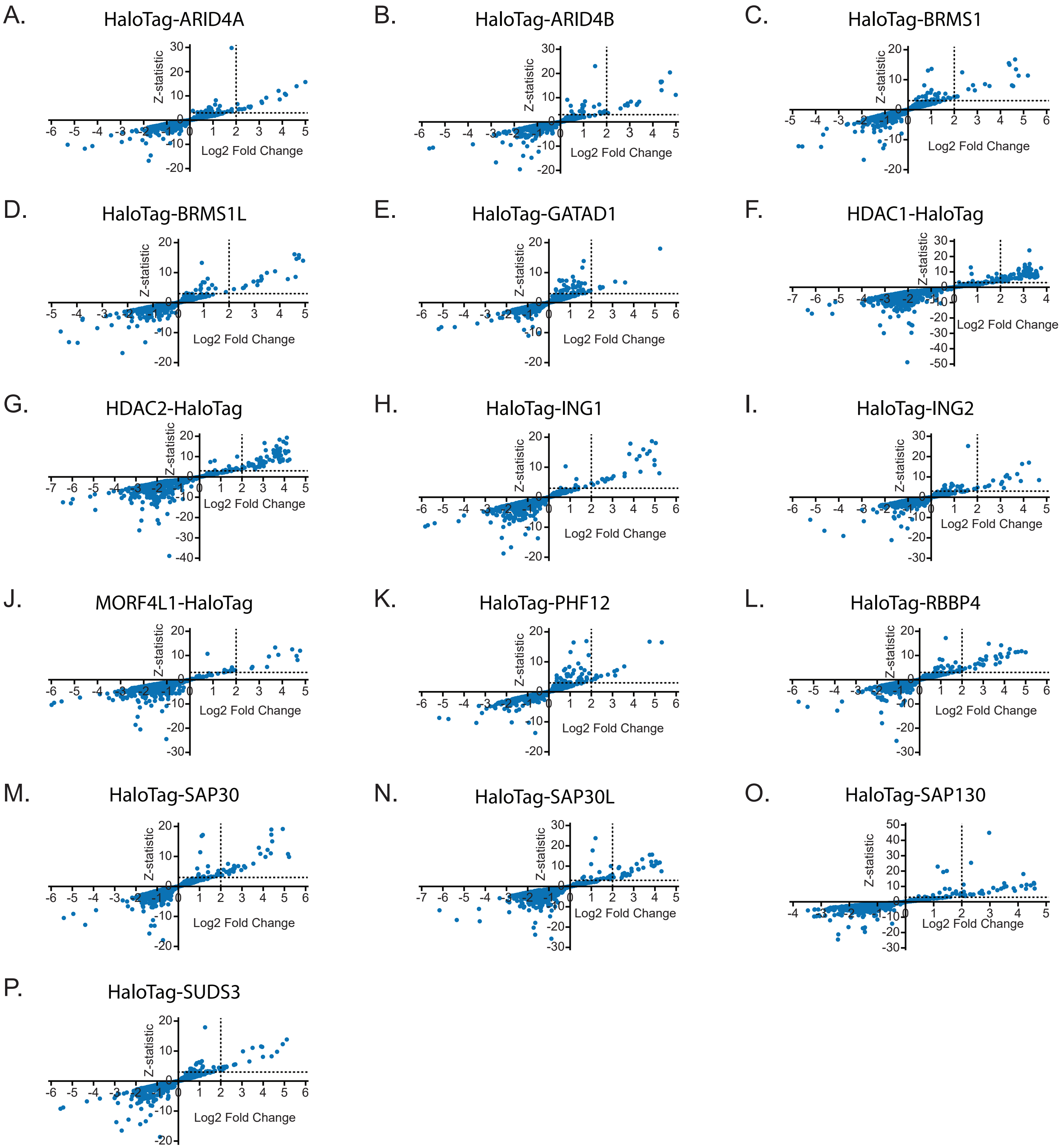

Supplementary Figure 6 | Adams et al.

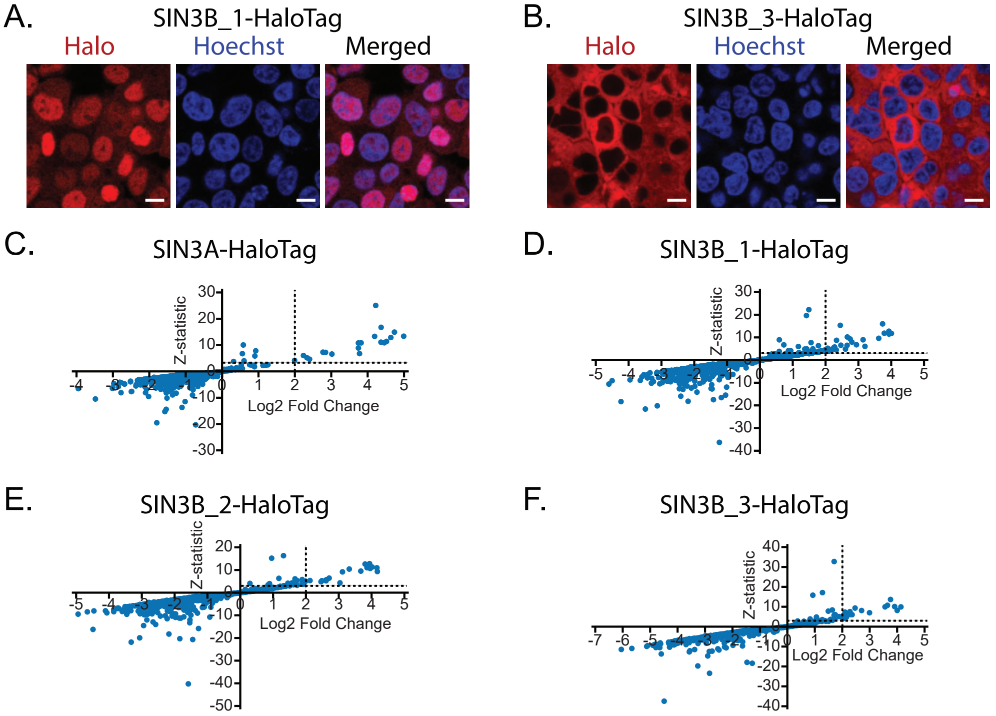

Supplementary Figure 7 | Adams et al.

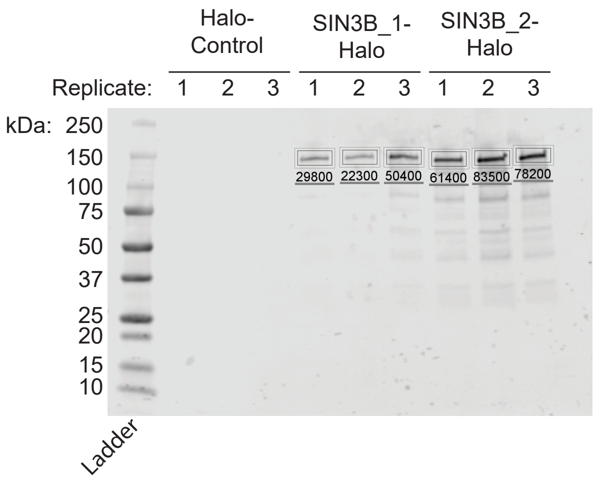

Supplementary Figure 8A | Adams et al.

```
         10        20        30        40        50        60
....|....|....|....|....|....|....|....|....|....|....|....|
GGTACCTGTAGTATAATTGTACAAGAGAGAGAGAGCAGAGAGAAGGGTCAGAAGAGGCCA 60

         70        80        90       100       110       120
....|....|....|....|....|....|....|....|....|....|....|....|
AGTGATGGAAATAGTGGATTAATGGCAAAAAAGCAAAAGCGTACCCCAAAGCGAACAAGT 120

        130       140       150       160       170       180
....|....|....|....|....|....|....|....|....|....|....|....|
GCTGCAGCCAAAAATGAAAAGAATGGAACAGGACAAAGCAGTGATAGTGAAGATCTTCCT 180

        190       200       210       220       230       240
....|....|....|....|....|....|....|....|....|....|....|....|
GTCCTAGACAATTCAAGTAAATGTACCCCAGTAAAGCATCTTAATGTATCTAAGCCACAG 240

        250       260       270       280       290       300
....|....|....|....|....|....|....|....|....|....|....|....|
AAACTTGCACGATCTCCTGCAAGAATATCCCCGCACATCAAAGATGGAGAGAAAGATAAA 300

        310       320       330       340       350       360
....|....|....|....|....|....|....|....|....|....|....|....|
CACAGAGAAAAACATCCGAATTCATCCCCTAGGACATATAAATGGTCCTTCCAATTGAAC 360

        370       380       390       400       410       420
....|....|....|....|....|....|....|....|....|....|....|....|
GAACTCGATAACATGAACAGTACAGAACGGATCAGCTTCCTGCAGGAGAAACTGCAGGAG 420

        430       440       450       460       470       480
....|....|....|....|....|....|....|....|....|....|....|....|
ATTAGGAAGTATTACATGAGCTTGAAGTCCGAGGTCGCAACAATCGATCGCAGAAGGAAG 480

        490       500       510       520       530       540
....|....|....|....|....|....|....|....|....|....|....|....|
CGCCTGAAAAAGAAGGACAGGGAAGTGTCTCATGCGGGAGCCTCCATGTCATCTGCTTCA 540

        550       560       570       580       590       600
....|....|....|....|....|....|....|....|....|....|....|....|
TCAGACACTGGAATGAGTCCCTCATCATCATCTCCCCCACAAAATGTACTTGCTGTAGAA 600

        610       620       630
....|....|....|....|....|....|....|
TGCAGGTGAGTTTAAACGAATTCGGGCTCGGTACC 635
```

Supplementary Figure 8B | Adams et al.

```
         10        20        30        40        50        60
....|....|....|....|....|....|....|....|....|....|....|....|
AAAAAGCGATCGCCATGCCCTTGGGGCTGAAACCTACATGTAGTGTATGCAAGACTACGT 60

         70        80        90       100       110       120
....|....|....|....|....|....|....|....|....|....|....|....|
CATCCAGCATGTGGAAGAAAGGTGCCCAAGGGGAAATTCTGTGCCATCACTGTACAGGGC 120

        130       140       150       160       170       180
....|....|....|....|....|....|....|....|....|....|....|....|
GAGGCGGAGCTGGCTCAGGTGGTGCTGGATCCGGTGCAGCCGGGGGCACCGGTGGATCAG 180

        190       200       210       220       230       240
....|....|....|....|....|....|....|....|....|....|....|....|
GAGGCGGGGGGTTCGGAGCGGCCACCTTCGCGTCTACCAGTGCGACTCCGCCGCAGTCTA 240

        250       260       270       280       290       300
....|....|....|....|....|....|....|....|....|....|....|....|
ACGGCGGGGGTGGGGGGAAACAGTCTAAGCAAGAGATCCATAGACGATCTGCACGATTGC 300

        310       320       330       340       350       360
....|....|....|....|....|....|....|....|....|....|....|....|
GAAATACTAAGTACAAATCAGCACCTGCCGCTGAGAAAAAAGTCTCCACTAAAGGTAAAG 360

        370       380       390       400       410       420
....|....|....|....|....|....|....|....|....|....|....|....|
GGCGGCGCCATATTTTTAAGCTCAAAAATCCCATCAAAGCACCAGAATCCGTCTCCACCA 420

        430       440       450       460       470       480
....|....|....|....|....|....|....|....|....|....|....|....|
TAATCACGGCTGAGTCCATCTTCTACAAAGGAGTTTATTACCAAATTGGCGACGTTGTTT 480

        490       500       510       520       530       540
....|....|....|....|....|....|....|....|....|....|....|....|
CTGTGATAGATGAACAGGACGGCAAGCCATACTACGCTCAAATACGGGGGTTCATACAGG 540

        550       560       570       580       590       600
....|....|....|....|....|....|....|....|....|....|....|....|
ATCAATACTGCGAGAAGTCTGCGGCACTGACTTGGCTTATTCCAACGCTCTCCTCCCCGC 600

        610       620       630       640       650       660
....|....|....|....|....|....|....|....|....|....|....|....|
GAGACCAGTTTGATCCCGCCTCATACATTATAGGGCCTGAGGAGGATCTGCCAAGAAAAA 660

        670       680       690       700       710       720
....|....|....|....|....|....|....|....|....|....|....|....|
TGGAATACCTGGAATTTGTCTGTCACGCCCCCTCCGAATATTTCAAGTCAAGGTCTTCCC 720

        730       740       750       760       770       780
....|....|....|....|....|....|....|....|....|....|....|....|
CCTTTCCAACCGTCCCGACGCGGCCTGAAAAAGGGTACATATGGACCCATGTGGGTCCGA 780

        790       800       810       820       830
....|....|....|....|....|....|....|....|....|....|....|..
CGCCTGCTATCACAATTAAAGAATCAGTAGCGAATCACCTTTAGGTTTAAACAAAAA 837
```

Supplementary Figure 8C | Adams et al.

```
         10        20        30        40        50        60
....|....|....|....|....|....|....|....|....|....|....|....|
ATGGCATATAGCCAAGGTGGGGGCAAAAAAAAAGTATGTTATTATTATGATGGAGATATC 60

         70        80        90       100       110       120
....|....|....|....|....|....|....|....|....|....|....|....|
GGTAACTATTACTATGGTCAGGGCCACCCAATGAAGCCACATAGAATCAGGATGACTCAC 120

        130       140       150       160       170       180
....|....|....|....|....|....|....|....|....|....|....|....|
AACCTGCTGTTGAATTATGGGTTGTATCGAAAGATGGAGATCTATAGACCACATAAGGCC 180

        190       200       210       220       230       240
....|....|....|....|....|....|....|....|....|....|....|....|
ACTGCTGAAGAGATGACCAAGTATCATTCTGATGAGTATATTAAATTTCTGAGGTCCATC 240

        250       260       270       280       290       300
....|....|....|....|....|....|....|....|....|....|....|....|
AGGCCAGACAACATGAGTGAGTACAGTAAACAAATGCAGCGCTTTAATGTGGGTGAGGAT 300

        310       320       330       340       350       360
....|....|....|....|....|....|....|....|....|....|....|....|
TGCCCAGTGTTCGACGGTCTGTTTGAGTTCTGTCAGCTCAGCACTGGCGGCTCTGTTGCA 360

        370       380       390       400       410       420
....|....|....|....|....|....|....|....|....|....|....|....|
GGGGCGGTAAAACTTAACCGCCAGCAAACGGATATGGCTGTTAATTGGGCAGGTGGCTTG 420

        430       440       450       460       470       480
....|....|....|....|....|....|....|....|....|....|....|....|
CATCATGCGAAAAAGTCCGAGGCTAGTGGGTTTTGTTACGTCAACGATATTGTCTTGGCC 480

        490       500       510       520       530       540
....|....|....|....|....|....|....|....|....|....|....|....|
ATCCTTGAGTTGCTCAAATACCATCAGCGCGTTCTCTACATAGATATCGATATCCATCAC 540

        550       560       570       580       590       600
....|....|....|....|....|....|....|....|....|....|....|....|
GGTGACGGCGTGGAAGAGGCCTTTTACACAACAGACAGAGTGATGACGGTTTCCTTCCAC 600

        610       620       630       640       650       660
....|....|....|....|....|....|....|....|....|....|....|....|
AAGTATGGCGAGTATTTCCCTGGCACTGGCGATCTCCGCGACATCGGTGCGGGTAAAGGA 660

        670       680       690       700       710       720
....|....|....|....|....|....|....|....|....|....|....|....|
AAGTACTACGCGGTTAACTTCCCCATGCGAGACGGGATAGACGACGAGTCCTATGGCCAA 720

        730       740       750       760       770       780
....|....|....|....|....|....|....|....|....|....|....|....|
ATATTTAAGCCCATAATATCTAAAGTGATGGAAATGTATCAACCGAGCGCCGTGGTTTTG 780

        790       800       810       820       830       840
....|....|....|....|....|....|....|....|....|....|....|....|
CAGTGCGGGGCAGATAGTTTGTCTGGTGATCGCCTTGGGTGCTTTAATCTTACCGTCAAA 840

        850       860       870       880       890       900
....|....|....|....|....|....|....|....|....|....|....|....|
GGTCACGCCAAGTGCGTAGAAGTAGTCAAGACATTCAACTTGCCTCTCCTCATGTTGGGG 900

        910       920       930       940       950       960
....|....|....|....|....|....|....|....|....|....|....|....|
GGCGGCGGGTACACGATCAGAAATGTGGCTAGATGTTGGACGTATGAAACCGCAGTTGCG 960
```

Supplementary Figure 8C | Adams et al.

```
        970       980       990      1000      1010      1020
....|....|....|....|....|....|....|....|....|....|....|....|
CTGGATTGCGAGATTCCAAATGAGTTGCCTTACAACGATTATTTTGAATATTTTGGCCCG 1020

       1030      1040      1050      1060      1070      1080
....|....|....|....|....|....|....|....|....|....|....|....|
GATTTCAAACTTCATATTTCCCCTAGTAATATGACTAACCAAAATACCCCGGAATATATG 1080

       1090      1100      1110      1120      1130      1140
....|....|....|....|....|....|....|....|....|....|....|....|
GAAAAAATAAAACAGCGACTCTTCGAGAATCTTCGGATGTTGCCCCATGCTCCCGGCGTG 1140

       1150      1160      1170      1180      1190      1200
....|....|....|....|....|....|....|....|....|....|....|....|
CAGATGCAGGCGATACCAGAGGATGCGGTCCACGAGGACAGCGGAGATGAAGATGGTGAG 1200

       1210      1220      1230      1240      1250      1260
....|....|....|....|....|....|....|....|....|....|....|....|
GACCCGGATAAGCGAATATCCATTCGCGCCAGTGATAAACGGATAGCCTGTGACGAAGAA 1260

       1270      1280      1290      1300      1310      1320
....|....|....|....|....|....|....|....|....|....|....|....|
TTTTCTGACAGTGAAGATGAGGGAGAGGGAGGCCGCAGGAATGTGGCTGACCATAAAAAG 1320

       1330      1340      1350      1360      1370      1380
....|....|....|....|....|....|....|....|....|....|....|....|
GGCGCGAAGAAAGCAAGAATTGAAGAGGACAAAAAGGAAACAGAGGACAAGAAGACTGAT 1380

       1390      1400      1410      1420      1430      1440
....|....|....|....|....|....|....|....|....|....|....|....|
GTTAAGGAGGAGGACAAGTCAAAAGACAACAGTGGTGAGAAAACAGATACTAAAGGAACT 1440

       1450      1460
....|....|....|....|....
AAATCTGAGCAGCTCTCTAATCCG 1464
```

Supplementary Figure 8D | Adams et al.

```
        10        20        30        40        50        60
....|....|....|....|....|....|....|....|....|....|....|....|
AAAAGCGATCGCCATGGCACCGAAACAAGACCCAAAACCCAAGTTCCAAGAAGGGGAGAG 60

        70        80        90       100       110       120
....|....|....|....|....|....|....|....|....|....|....|....|
GGTGTTGTGTTTCCACGGACCACTGTTGTATGAGGCCAAATGCGTCAAGGTAGCAATCAA 120

       130       140       150       160       170       180
....|....|....|....|....|....|....|....|....|....|....|....|
AGATAAACAGGTTAAATACTTCATTCACTATTCTGGCTGGAATAAGAATTGGGATGAGTG 180

       190       200       210       220       230       240
....|....|....|....|....|....|....|....|....|....|....|....|
GGTGCCGGAGAGCAGAGTTCTGAAATATGTAGATACCAATTTGCAAAAGCAACGGGAACT 240

       250       260       270       280       290       300
....|....|....|....|....|....|....|....|....|....|....|....|
CCAGAAGGCAAATCAGGAACAATATGCAGAAGGGAAGATGAGGGGAGCAGCGCCGGGAAA 300

       310       320       330       340       350       360
....|....|....|....|....|....|....|....|....|....|....|....|
AAAAACCTCTGGACTTCAGCAAAAAAACGTGGAGGTCAAGACTAAAAAGAACAAGCAGAA 360

       370       380       390       400       410       420
....|....|....|....|....|....|....|....|....|....|....|....|
GACGCCTGGCAACGGAGATGGGGGCTCCACGTCTGAAACGCCACAGCCCCCAAGGAAAAA 420

       430       440       450       460       470       480
....|....|....|....|....|....|....|....|....|....|....|....|
ACGGGCCCGCGTTGACCCCACTGTAGAAAACGAAGAGACGTTCATGAATCGAGTCGAGGT 480

       490       500       510       520       530       540
....|....|....|....|....|....|....|....|....|....|....|....|
GAAAGTCAAGATCCCAGAAGAGTTGAAGCCTTGGCTTGTCGACGATTGGGACCTCATAAC 540

       550       560       570       580       590       600
....|....|....|....|....|....|....|....|....|....|....|....|
CAGACAAAAACAACTCTTCTATCTCCCTGCGAAGAAGAACGTTGATAGCATATTGGAGGA 600

       610       620       630       640       650       660
....|....|....|....|....|....|....|....|....|....|....|....|
TTATGCCAATTACAAAAAAAGCCGGGGTAATACCGATAACAAAGAGTATGCGGTTAATGA 660

       670       680       690       700       710       720
....|....|....|....|....|....|....|....|....|....|....|....|
GGTAGTAGCTGGAATTAAGGAATATTTCAACGTAATGCTCGGTACACAACTGCTGTACAA 720

       730       740       750       760       770       780
....|....|....|....|....|....|....|....|....|....|....|....|
ATTTGAGCGCCCGCAATATGCGGAGATTCTTGCTGACCATCCAGATGCACCGATGAGTCA 780

       790       800       810       820       830       840
....|....|....|....|....|....|....|....|....|....|....|....|
AGTATATGGTGCTCCGCACCTGTTGCGGCTGTTTGTTCGGATCGGGGCAATGTTGGCATA 840

       850       860       870       880       890       900
....|....|....|....|....|....|....|....|....|....|....|....|
TACACCACTGGACGAGAAAAGCTTGGCCTTGCTGTTGAACTACCTTCACGACTTCCTGAA 900

       910       920       930       940       950       960
....|....|....|....|....|....|....|....|....|....|....|....|
ATACCTGGCAAAGAACAGTGCAACTCTGTTCTCTGCCAGTGACTACGAGGTCGCTCCTCC 960
```

Supplementary Figure 8D | Adams et al.

```
        970       980       990
....|....|....|....|....|....|....|..
CGAGTATCATCGAAAAGCTGTGTGAGTTTAAACAAAA 997
```

Supplementary Figure 8E | Adams et al.

```
        10        20        30        40        50        60
....|....|....|....|....|....|....|....|....|....|....|....|
ATGCATCCTGCAAGCGCATAGGATCCAGCTACCGGGCACTCCCCAAAACCTACCAGCAGC 60

        70        80        90       100       110       120
....|....|....|....|....|....|....|....|....|....|....|....|
CCAAGTGCAGTGGGAGGACAGCCATCTGCAAGGAGCTTGACCATTGGACACTTCTCCAGG 120

       130       140       150       160       170       180
....|....|....|....|....|....|....|....|....|....|....|....|
GTTCGTGGACAGACGATTACTGCATGTCCAAGTTCAAGAATACCTGCTGGATTCCAGGAT 180

       190       200       210       220       230       240
....|....|....|....|....|....|....|....|....|....|....|....|
ATAGTGCAGGGGTACTGAACGACACCTGGGTCTCCTTCCCTTCCTGGTCTGAGGACTCCA 240

       250       260       270
....|....|....|....|....|....|
CGTTCGTCAGCTCCAAGAAGACACCGTACG 270
```

Supplementary Figure 8F | Adams et al.

```
        10        20        30        40        50        60
....|....|....|....|....|....|....|....|....|....|....|....|
AAAAGCGATCGCCATGCAGCGTCATTCACGGCATTTCCTCTTGGTGCAGGTACTGAACGA 60

        70        80        90       100       110       120
....|....|....|....|....|....|....|....|....|....|....|....|
CACCTGGGTCTCCTTCCCTTCCTGGTCTGAGGACTCCACGTTCGTCAGCTCCAAGAAGAC 120

       130
....|....|..
ACCGTACGAAAA 132
```

Supplementary Figure 8G | Adams et al.

```
         10        20        30        40        50        60
....|....|....|....|....|....|....|....|....|....|....|....|
AATTAAGAAGCGATCGCCATGCCGGAGCACAGCAGGAAGCGCTCCCGGCCCTCGCTCCTC

         70        80        90       100       110       120
....|....|....|....|....|....|....|....|....|....|....|....|
CGCCCCGTGTCTGCACCCGCCAAGAAAAAAATGAAACTTCGTGGTACCAAAGTTTAAACT


....|.
TGATAA
```
